# Difficult-to-neutralize global HIV-1 isolates are neutralized by antibodies targeting open envelope conformations

**DOI:** 10.1038/s41467-019-10899-2

**Published:** 2019-07-01

**Authors:** Qifeng Han, Julia A. Jones, Nathan I. Nicely, Rachel K. Reed, Xiaoying Shen, Katayoun Mansouri, Mark Louder, Ashley M. Trama, S. Munir Alam, Robert J. Edwards, Mattia Bonsignori, Georgia D. Tomaras, Bette Korber, David C. Montefiori, John R. Mascola, Michael S. Seaman, Barton F. Haynes, Kevin O. Saunders

**Affiliations:** 1 Department of Medicine, Duke University Medical Center, Durham, NC 27710 USA; 2 Duke Human Vaccine Institute, Duke University Medical Center, Durham, NC 27710 USA; 3 Department of Cell Biology, Duke University Medical Center, Durham, NC 27710 USA; 4 Vaccine Research Center, National Instiftute of Allergy and Infectious Diseases (NIAID), NIH, Bethesda, MD 20892 USA; 5 Department of Surgery, Duke University Medical Center, Durham, NC 27710 USA; 6 Department of Microbiology and Molecular Genetics, Duke University Medical Center, Durham, NC 27710 USA; 7 Department of Immunology, Duke University Medical Center, Durham, NC 27710 USA; 8 Los Alamos National Laboratory, Los Alamos, NM 87545 USA; 9 Center for Virology and Vaccine Research, Israel Deaconess Medical Center, Boston, MA 02115 USA

**Keywords:** Adaptive immunity, HIV infections, Vaccines

## Abstract

The HIV-1 envelope (Env) is the target for neutralizing antibodies and exists on the surface of virions in open or closed conformations. Difficult-to-neutralize viruses (tier 2) express Env in a closed conformation antigenic for broadly neutralizing antibodies (bnAbs) but not for third variable region (V3) antibodies. Here we show that select V3 macaque antibodies elicited by Env vaccination can neutralize 26% of otherwise tier 2 HIV-1 isolates in standardized virus panels. The V3 antibodies only bound to Env in its open conformation. Thus, Envs on tier 2 viruses sample a state where the V3 loop is not in its closed conformation position. Envelope second variable region length, glycosylation sites and V3 amino acids were signatures of neutralization sensitivity. This study determined that open conformations of Env with V3 exposed are present on a subset of otherwise neutralization-resistant virions, therefore neutralization of tier 2 HIV-1 does not always indicate bnAb induction.

## Abstract (web summary)

Here, the authors report that specific monoclonal antibodies isolated from vaccinated rhesus macaques can neutralize a subset of Tier 2 difficult-to-neutralize HIV-1 that express Env in an open conformation, suggesting that V3 loop-specific targeting can bias the estimation of vaccine-induced bnAbs.

## Introduction

The human immunodeficiency virus subtype 1 (HIV-1) envelope (Env) protein is a heterodimeric trimer consisting of gp120 and gp41 subunits^1^. The gp120 is divided into five conserved (C1–C5) regions and five variable loop (V1–V5) regions based on primary amino acid sequence^2^. The gp120 engages its receptor CD4, afterwhich, it undergoes conformational changes that expose variable loop regions and the coreceptor binding site on an open Env conformation^3,4^. There are many Env epitopes that confer antibody neutralization of HIV-1 with open Env conformations (tier 1 viruses), and during natural infection, antibodies targeting these epitopes dominate the antibody response^5^. The third variable region (V3) is among the most immunogenic regions on Env^6,7^, and is relatively conserved compared to other hypervariable regions^8^. However, antibodies specific for the V3 region have not been broadly neutralizing^9,10^, presumably because the V3 region is not accessible on HIV-1 Env prior to conformational changes induced by CD4 engagement^11–13^. Unliganded HIV-1 Env structures showing a hidden V3 loop are consistent with this notion^4,13,14^. Furthermore, this hypothesis is supported by the enhanced HIV-1 neutralization by V3-specific antibodies in the presence of soluble CD4^11,12^. In the absence of CD4, mutations in the hydrophobic core of the gp120 subunit or at the N301 and N160 glycosylation sites can render the V3 region accessible for V3-specific neutralizing antibodies^15^. Thus, amino acid sequence or glycosylation changes are sufficient for V3 region exposure on Env in the absence of CD4 binding^16^.

HIV-1 Env is the sole target for HIV-1 broadly neutralizing antibodies (bnAbs)^17^. To understand the significance of neutralization of diverse HIV-1 isolates, HIV-1 isolates have been typed into tiers based on their sensitivity to polyclonal neutralizing antibodies in sera from HIV-1-infected individuals^18,19^. Viruses have been typed as tier 1A, 1B, 2, or 3, with 1A being the most sensitive and 3 being the most resistant^18^. Viruses within tier 2 include primary, circulating viruses whereas tier 1 viruses include many laboratory-adapted HIV-1 strains^20^. Thus, induction of tier 2 neutralization is a current goal for immune responses induced by antibody-based vaccines^19^. Single molecule fluorescence energy transfer (smFRET) analyses suggest the more difficult-to-neutralize viruses express Env in a closed conformation distinct from that of neutralization-sensitive tier 1 viruses^21^. This native, closed conformation is recognized by bnAbs, but not by coreceptor binding site antibodies^21^, which require CD4-mediated Env activation^22^. Presently, only 8 known categories of neutralizing epitopes on HIV-1 Env confer broad neutralization^23^.

Here, we have isolated from Env-vaccinated macaques three new V3 peptide-specific monoclonal antibodies. These three vaccine-elicited antibodies neutralized a subset of tier 2 HIV-1 isolates in standardized HIV-1 virus panels. Crystal structures of two of the antibodies revealed that one antibody approached the V3 loop head-on, while the other antibody approached the V3 loop from the side. Molecular modeling of V3 peptide:antibody crystal structures onto Env trimers and surface plasmon resonance with soluble Env trimers determined that these V3 antibodies were only capable of binding CD4-triggered, open conformations of HIV-1 Env. Computational analyses of the neutralization sensitivity of approximately 300 HIV-1 isolates to these V3 antibodies defined second variable region length, amino acids within the V3 region, and multiple Env glycosylation sites as signatures of tier 2 virus neutralization susceptibility to V3 antibodies. These results demonstrate that a subset of tier 2 viruses have accessible V3 regions on virions that vaccine-elicited antibodies can target. Thus, V3 loop-specific antibody neutralization of tier 2 viruses can confound the determination of vaccine induction of bnAbs based on current tier 2 virus neutralization panels.

## Results

### Induction of heterologous tier 2 HIV neutralizing antibodies

**Fig. 1:**
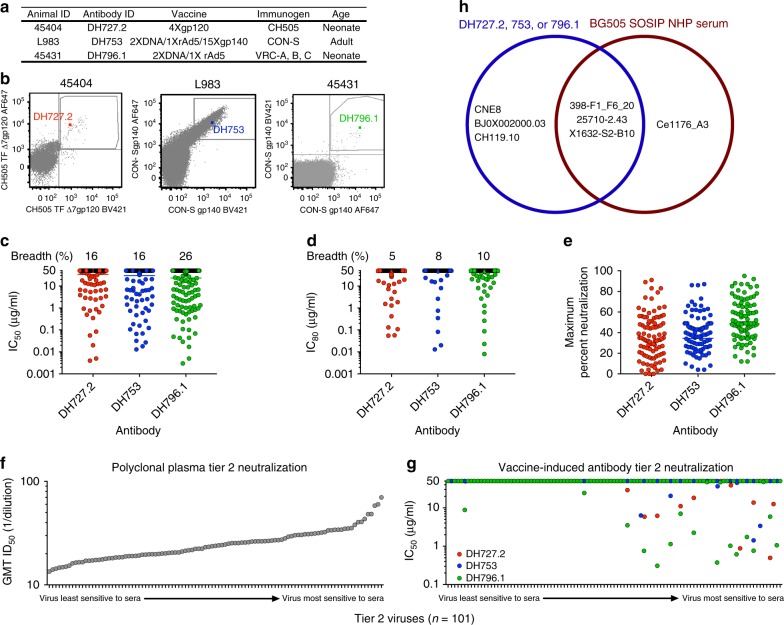
Vaccine-induced antibodies neutralize tier 2 viruses. **a** Summary of the vaccine regimens and macaque demographics from which the three antibodies DH727.2, DH753, and DH796.1 were isolated. **b** Antigen-specific single B cell sorting of vaccinated macaque PBMCs. Antigen-specific single B cells were isolated with fluorophore-labeled CH505 gp120 or CON-S gp140 as indicated on the *x* and *y*-axes. The B cell encoding the antibody of interest is shown as a colored square in the FACS plot. **c**, **d** *In vitro* antibody neutralization of HIV-1 infection of TZM-bl cells. Each symbol signifies one of the 292 HIV-1 isolates tested. Neutralization titers are represented as the concentration in µg mL^‒1^ that inhibits **c** 50% or **d** 80% of virus replication (IC50 or IC80 respectively). SIVmac239 and MuLV were used as negative controls and titers for both viruses were > 50 µg mL^‒1^. Horizontal bar indicates the geometric mean neutralization titer of all isolates. The neutralization breadth is shown above each column as the percentage of viruses neutralized by 50% **c** or 80% **d**. **e** Maximum percent neutralization achieved by each vaccine-elicited antibody against 93 early/acute tier 2 clade C HIV-1 isolates. Each symbol signifies one HIV-1 isolate. Horizontal bar indicates the mean maximum percent neutralization. **f**, **g** Vaccine-induced antibodies preferentially neutralize the most sensitive tier 2 viruses. Polyclonal plasmas from HIV-1 infected individuals were used to phenotype 101 HIV-1 isolates as tier 2 viruses **f**. The plasma dilution that inhibited 50% of virus replication (ID50) for each virus is shown for all serum samples as the geometric mean titer (GMT). The viruses are ordered according to increasing sensitivity to the polyclonal sera neutralizing antibodies. **g** Vaccine-induced antibodies DH727.2 (red), DH753 (blue), and DH796.1 (green) neutralization titers as IC50 in µg mL^‒1^ for the 101 tier 2 HIV-1 isolates shown in **f**. **h** Venn diagram showing the overlap of viruses from the 12-virus global panel that were neutralized by DH727.2, DH753, DH796.1 isolated from vaccination and macaque immune sera from monkeys immunized with BG505 SOSIPs^29^. Five of the viruses were not neutralized by DH727.2, DH753, DH796.1 or BG505 SOSIP-immune sera. Source data are provided as a Source Data file

In three separate studies, Indian origin rhesus macaques were immunized with either CH505 gp120; unstabilized, uncleaved CON-S gp140 protein and gp145 DNAs^24^; or unstabilized, cleaved VRC-A, B, and C gp160 HIV-1 envelopes^25^ (Fig. 1a). To characterize the antibody response in these studies, we sorted blood single B cells that bound fluorophore-labeled HIV-1 envelope (Supplementary Fig. 1). Monoclonal antibody DH727.2 was isolated from a neonatal macaque immunized with CH505 gp120 (Fig. 1b). DH753 was isolated from a group M consensus CON-S envelope-immunized adult macaque (Fig. 1b). Lastly, DH796.1 was isolated from a neonatal macaque immunized with VRC-A, B, and C Envs^25^. The three antibodies utilized distinct VH and JH gene segments and different HCDR3 lengths ranging from 10 to 13 amino acids (Supplementary Table 1). In contrast, both DH727.2 and DH796.1 used Vκ 2-S20*01 and Jκ 3–2*1 gene segments and 9 amino acid LCDR3s. DH753 differed from the other two antibodies as it was derived from Vκ 1-F and Jκ 1-LC1 gene segments and encoded an 11 amino acid LCDR3 (Supplementary Table 1).

We tested the ability of these vaccine-elicited antibodies to neutralize tier 2 HIV-1 viruses in four standardized panels of viruses—multiclade 30-virus panel, early/acute clade C 100-virus panel (composed of 7 tier 1 and 93 tier 2 viruses), the global 12-virus panel, and a global 208-virus panel (Supplementary Fig. 2)^26,27^. There was overlap of viruses among the panels, such that there was a total of 292 unique tier 2 HIV-1 pseudoviruses tested. The antibody DH796.1 neutralized significantly more viruses (26% of the 292 viruses) than DH727.2 and DH753 as determined by IC50 titers (Fig. 1c, Supplementary fig. 3A and Supplementary Data 1; Fisher’s exact test *p* = 0.005 for DH796.1 compared to DH753 and *p* = 0.003 when compared to DH727.2, *n* = 292). The neutralization breadth of DH796.1 decreased to 10% when neutralization was considered as inhibition of 80 percent of virus replication (IC80; Fig. 1d and Supplementary Data 1). Neutralization potency determined as geometric mean IC50 was modest for all three antibodies against all 292 viruses (DH796.1 = 2.6, DH727.2 = 4.6, DH753 = 2.1 μg mL^−1^), and among positive responses there were no significant differences in potency of the 3 antibodies (*p* > 0.05, Wilcoxon Rank sum test and Supplementary Fig. 1c, 2c, and 3a). None of the antibodies blocked CD4 binding to envelope, hence this was not the mechanism of neutralization (Supplementary Fig. 4).

In some cases, antibodies can neutralize a substantial proportion of the replicating virus, but do not eliminate all HIV-1 replication^28^. As a measure of complete neutralization we examined the maximum neutralization achieved against the tier 2 clade C early/acute viruses. For the 93 tier 2 viruses in this panel, incomplete neutralization was most evident for DH753, where 18 tier 2 early/acute clade C viruses were neutralized greater than 50% but none of the viruses were neutralized above 90% **(**Fig. 1e). DH796.1 and DH727.2 inhibited virus infection by 90% or greater for only two and one virus respectively **(**Fig. 1e). Thus, these antibodies lacked complete neutralization against most early/acute clade C viruses tier 2 HIV-1 isolates.

Although viruses are categorized into tiers, the sensitivity of the viruses within a tier still varies creating a spectrum of antibody neutralization sensitivity^18,26^. We examined their neutralization potency against 101 tier 2 viruses with known sensitivities to 205 HIV-1-infected polyclonal sera^26^. The 101 tier 2 viruses demonstrated a range of sensitivities to the HIV-1-infected polyclonal sera, which allowed them to be ranked according to differences in the geometric mean inhibitory dilution 50 (ID50) titer for all 205 plasma samples (Fig. 1f and Supplementary Data 2). The tier 2 viruses with detectable IC50s for DH727.2, DH753, or DH796.1 tended to be at the most sensitive end of the neutralization spectrum (Fig. 1f). There were 21 viruses with detectable IC50 values and 18 of these 21 isolates were at the most sensitive end of the tier 2 virus spectrum (Fig. 1f and Supplementary Data 2). Therefore, the tier 2 viruses that were the most resistant to the 205 polyclonal sera were also the most resistant to the vaccine induced V3 antibodies (Fig. 1f and Supplementary Data 2). Thus, tier 2 isolates possessed distinct susceptibility to V3 antibody neutralization based on which end of the neutralization sensitivity spectrum they resided.

BG505 SOSIP trimer immunization has been reported to elicit serum neutralization activity in macaques against X1632-S2-B10, Ce1176_A3, 25710-2.43, and 398-F1_F6_20 in the 12-virus global panel^29^. We assessed whether our three macaque neutralizing antibodies could neutralize the same isolates in the 12-virus panel. X1632-S2-B10, CNE8, and 398-F1_F6_20 were sensitive to 2 of 3 macaque antibodies, and 25710-2.43 was sensitive to all three antibodies (Supplementary Fig. 3b). Similarly, 25710-2.43 and CNE8 were sensitive to 3074, a human V3 antibody from natural infection (Supplementary Fig. 3b). Although none of the antibodies potently neutralized these viruses, three HIV-1 isolates (398-F1_F6_20, 25710-2.43, and X1632-S2-B10) were found to be neutralized by both DH796.1 and BG505 induced macaque sera^29^ (Fig. 1h). DH796.1 differed from the BG505 SOSIP immune sera in that it did not neutralize Ce1176_A3, but instead neutralized CNE8, BJ0X002000.03, and CH119.10. Thus, 3 of 4 viruses sensitive to BG505 SOSIP immune sera were also sensitive to our 3 vaccine-elicited macaque V3 antibodies.

### HIV-1 clade-specific antibody neutralization activity

**Fig. 2:**
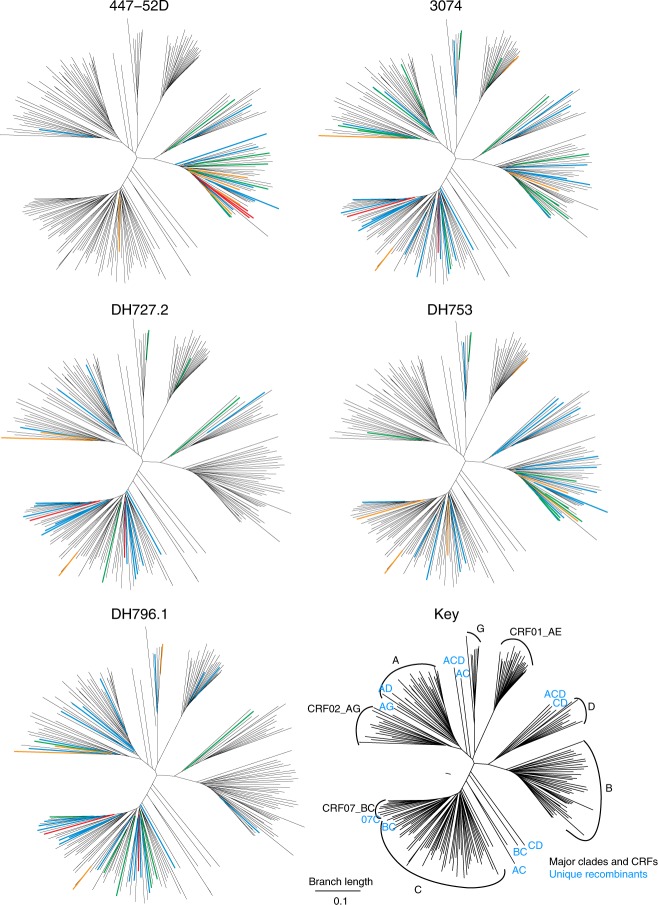
HIV-1 phylogenetic trees indicate clade-specific antibody neutralization patterns. These phylogenetic trees are based on the 208 pseudovirus panel, to allow direct visual comparisons of the vaccine-elicited antibodies with 447-52D. The HIV-1 envelope gp160 sequences were used to construct maximum likelihood trees, each HIV-1 clade is indicated in the key. Circulating recombinant forms (CRFs)^30^ are indicated when they are major epidemic lineages: CRF02 is an AG recombinant that is A-like in Env and common in West Africa, CRF01 is an AE recombinant that is E-like in Env and common in southeast Asia, and CRF07 is a BC recombinant that is C-like in Env that is common in China. Unique recombinants are indicated in the dendrogram with blue letters indicating the parental virus clades. Neutralization IC50 titers for HIV-1 infection of TZM-bl cells are shown for two HIV-1 antibodies from natural infection (447-52D and 3074; top row) and vaccine-induced antibodies (DH727.2, DH753, DH796.1; bottom row). Neutralization potency is color-coded based on IC50 in µg mL^‒1^ as red 0.001–0.01, orange 0.01–0.1, green 0.1–1, blue 1–50 and black > 50. Of note is both the clade specificity of these antibodies, and the recurrence of the same subsets of viruses being sensitive within each clade. Source data are provided as a Source Data file

We examined differences in antibody neutralization across HIV-1 clades for the 208 viruses in the large global panel (Supplementary Fig. 2a). DH753 neutralized a subset of clade B viruses, while clade B viruses were almost completely resistant to DH727.2 and DH796.1 (Fig. 2). Conversely, clade C viruses were neutralized by all 3 antibodies (Fig. 2). Some clades were rarely neutralized, or only weakly, by any of the 3 antibodies, including clades A and D and the circulating recombinant form CRF01 (Fig. 2)^30^. Clade preferences were also evident for two antibodies with moderate neutralization breadth from infection (447-52D^31^ and 3074^32^). The antibodies tended to show a preference for the clade of the infecting or vaccine virus that stimulated the response. 447-52D, isolated from a person with a clade B infection, was specific for clade B viruses—driven by the clade B GPGR motif that is common only in clade B^31^ (Fig. 2). Antibody 3074, isolated from a person with a Circulating Recombinant Form 02 (CRF02)^30^ infection (an AG recombinant which is mostly clade A in Env), had relatively strong CRF02 responses, as well as additional responses. DH727.2 elicited by a clade C vaccine, CH505, favored clade C and CRF07 responses (a BC recombinant that is mostly clade C in Env). DH796.1, elicited by a clade A, B, and C trivalent vaccine favored A and C responses, and DH753, elicited by the CON-S vaccine that is central to the M group, reacted with both clade B and C viruses (Fig. 2). Examination of each branch shows that many of the viruses that were sensitive to 3074 were also sensitive to the vaccine-induced antibodies (Supplementary Data 1).

Since DH753 showed the ability to neutralize 15 of 41 clade B viruses (DH727 and DH796 did not recognize clade B viruses), we determined whether DH753 could neutralize a panel of clade B transmitted-founder (TF) viruses (Supplementary Fig. 3d). We found that all 21 clade B TF viruses were resistant to DH753, while 15/41 clade B viruses isolated from individuals in chronic infection were sensitive to DH753 (Fisher’s exact test, *n* = 21 and 41, *p* = 0.001). In contrast, 35/69 (51%) of TF viruses in the clade C panel were positive for IC50 neutralization by DH753 (Supplementary Fig. 3). Therefore, not all transmitted/founder viruses were resistant to DH753, and sensitivity appeared to have a degree of clade specificity.

### DH727.2, DH753 and DH796.1 bind to the gp120 V3 loop region

**Fig. 3:**
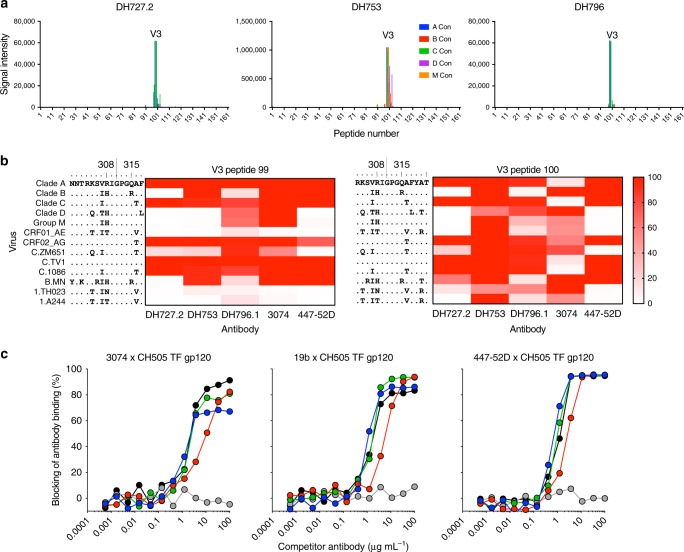
Tier 2 HIV-neutralizing antibodies bind linear V3 epitopes in HIV-1 gp120. **a** Antibody reactivity with a linear peptide array spanning the gp120 subunit of HIV-1 Env. Peptides from consensus sequences for clades A (blue), B (red), C (green), and D (purple) and group M (orange) HIV isolates are shown as different colors. Each graph represents the magnitude of binding to each peptide for each antibody. **b** Heatmap of cross-clade V3 peptide binding by different V3-specific antibodies. Binding to peptide number 99 (left) and 100 (right) are shown. Stronger binding is indicated by darker red colors. The sequences of the V3 peptides are shown on the left of the heatmap. HXB2 amino acid number is shown above the peptide sequences. Human monoclonal antibodies 3074 and 447–52D were included for comparison. **c** Macaque vaccine-induced antibody blocking of three human V3-specific HIV-1 antibodies binding to CH505 TF gp120. Each curve shows the mean percent blocking of Env binding by DH727.2 (blue), DH753 (red), or DH796.1 (green). Mean of 2 independent experiments is shown. As a positive control the human antibody (black) was used to block itself. An anti-influenza antibody (gray) was used as a negative control blocking antibody. Source data are provided as a Source Data file

To determine the Env epitopes recognized by the three macaque antibodies we used a HIV-1 linear peptide microarray^33^. Each of the three Abs showed specific binding only to the V3 (amino acids 298–321 by HxB2 numbering) region of gp120 (Fig. 3a). These antibodies bound to V3 peptides lacking glycans and thus were distinct from V3-glycan broadly neutralizing antibodies like PGT121. To determine the breadth of V3 peptide recognition we assessed binding by peptide array to two overlapping sets of V3 peptides (Fig. 3b). The peptides were derived from multiple clades, circulating recombinant forms of HIV-1, and common vaccine strains^33^. DH753 exhibited the broadest and highest magnitude V3 peptide reactivity but required amino acids 318–320 for optimal binding breadth (Fig. 3b, right). DH727.2 bound well to clade A and clade C V3 peptides—consistent with its neutralizing profile (Figs. 2 and 3b). The reduced binding breadth was associated with the presence of amino acid changes at positions 307 and 308 within the V3 loop (Fig. 3b). DH796.1 was also broadly reactive and binding was strongest when the peptide contained Arg308 (Fig. 3b). DH727.2 and human V3-specific antibody 447–52D shared similar binding reactivity patterns except for clade B peptides (Fig. 3b). The reactivity of 447–52D did not correspond to its neutralizing profile in that 447–52D is highly clade B specific (Figs. 2 and 3b). Human V3-specific antibody 3074 and DH753 were similar in that both antibodies required amino acids 318–320 for recognition of clade AE V3 peptides. Thr319 was favored for DH753 recognition (Fig. 3b), and macaque antibodies typically target the C-terminus of V3^34^. All three vaccine-induced antibodies blocked the binding of multiple V3 antibodies to Env (Fig. 3c).

### V3 antibody HIV-1 neutralization signatures

**Fig. 4:**
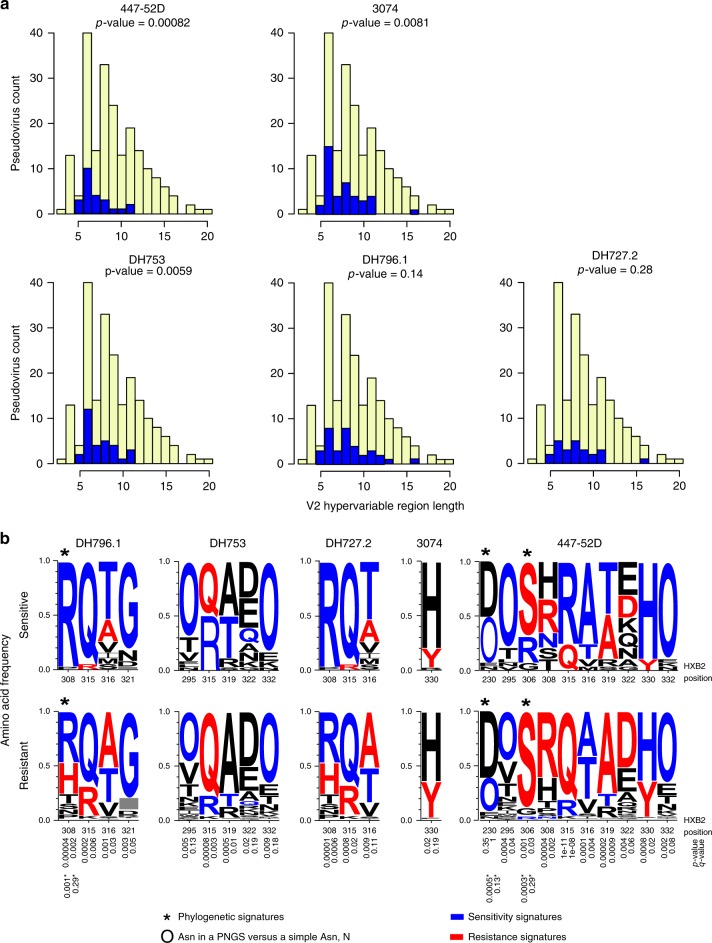
Signatures of V3 region-specific neutralization sensitivity. **a** V2 region length was associated with V3 antibody neutralization sensitivity. Bar graphs display the number of viruses with a given V2 hypervariable region length, and the colors indicate antibody neutralization resistance (yellow) and sensitivity (blue). Viruses were divided into two groups: a group with V2 lengths above 8 amino acids (the median) and a group with V2 lengths at the median or below. A Fisher’s exact test was used to compare the frequency of positive IC50 values in each group. *P*-values of < 0.006 withstand a Bonferroni correction (*n* = 47 viruses for DH727.2, *n* = 48 viruses for DH753, *n* = 77 viruses for DH796.1, *n* = 55 viruses for 3074, *n* = 24 viruses for 447–52D). Associations were significant for 447–52D and DH753. **b** Env signature patterns associated with V3 antibody neutralization. Sequence LOGOs show the frequency of amino acids at signature positions in the groups of viruses (*n* = 292) with detectable (sensitive) or undetectable (> 50 µg mL^‒1^; resistant) IC50 scores. Signature analysis was phylogenetically corrected and used an inclusive q-value cutoff to not miss signal. The 3 identified sites are indicated by asterisks. The *p* and q-values for these signatures are noted near the bottom of the figure, and are also indicated by an asterisk. We then used simple correlation analysis, and focused on identifying signatures in V3 region as they are most likely to be relevant by virtue of being in the contact region; sites included have a q-value < 0.2 and are in the V3 loop or are the glycosylation sites that frame it at positions 295 and 332 are included. The statistical support for these sites is noted just beneath the resistance LOGOs. “O” is indicative of an N in a glycosylation motif. Blue indicates amino acids in sites associated with sensitivity, red with resistance, and black was not significantly associated with either. Source data are provided as a Source Data file and Supplementary Data 1

To determine the virus attributes that contributed to V3 antibody neutralization sensitivity we examined the sequences of the more than 200 viruses tested for neutralization. First, we compared IC50 neutralization titer with length of, net charge of, and number of glycosylation sites within V1, V2, and V1 + V2 combined hypervariable regions. The V1 and V2 loop region was selected because of structural evidence showing that these loops may block access to the V3 loop when Env is in a closed conformation^4,13,14^. We divided the viruses into two groups, those with V2 lengths above the median and those at the median or below (the V2 length range was between 3 and 20, and the median was 8). Next, we used a Fisher’s exact test to compare the frequency of positive IC50 values in each group. 447-52D and DH753 neutralization showed a significant association with V2 length (*p* < 0.006 with Bonferroni correction), where pseudoviruses with long V2 loops were significantly more likely to be resistant (Fig. 4a). 3074 showed a supporting trend, and DH796.1 and DH727 were less well-powered and not significant (Fig. 4a). There was a weak trend suggesting that a few glycosylation sites in the V2 hypervariable region may also be associated with sensitivity (*p*-values of 0.01).

V3 region signatures patterns were specific for particular antibodies (Fig. 4b). Within the V3 loop, amino acids at positions 308, 315, 316 were associated with sensitivity to different antibodies. For example, Arg308 was required for DH796.1 and DH727 neutralization activity consistent with its requirement for binding in peptide microarrays (Figs. 3b and 4b). In contrast, Arg308 was associated with 447–52D resistance (Fig. 4b). In contrast, Arg315 was favored for 447-52D sensitivity, preferred for DH753 sensitivity, and most common in clade B envelope sequences (72%). Gln315 was most common in other clades and was strongly favored by both DH796.1 and DH727.2 (Fig. 4b). Thus, these signatures may be driving the distinctive clade specificities of these antibodies (Fig. 2). Potential N-linked glycosylation sites (PNGS) at the base of the V3 loop at positions 295 and 332 were enriched among viruses sensitive to DH753 and 447-52D. These glycosylation sites were located proximal to the Cys that form the disulfide bridge that closes the V3 loop, and may impact its orientation in an intact protein. A PNGS at position 230 was also enriched for 447–52D.

### Structural analysis of antibody binding to Env V3 peptides

**Fig. 5:**
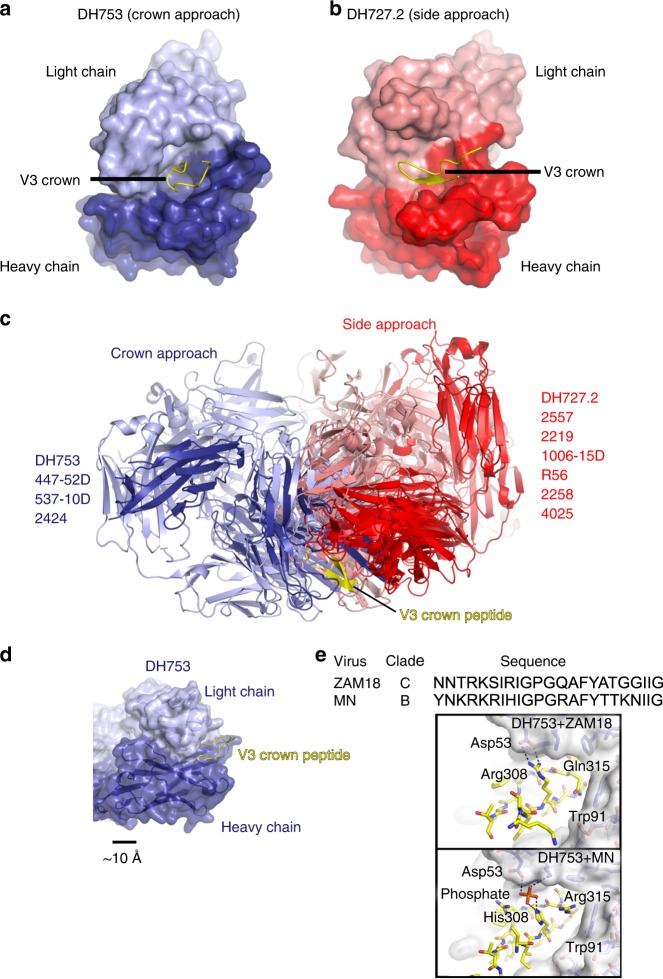
Structural analysis of macaque V3 antibodies. **a** The crystal structure of DH753 is shown with the L chain rendered in lilac, the FabH chain in dark purple, and bound ZAM18 V3 hairpin peptide in yellow. **b** The crystal structure of DH727.2 is shown in a similar orientation with the L chain in salmon, the FabH chain in red, and the ZAM18 V3 peptide again in yellow. **c** A superposition of several V3 antibody-peptide structures on the basis of the peptide (yellow) showed two clusterings. The ‘crown approach’ cluster included DH753 and other antibodies depicted in purple shades. The ‘side approach’ cluster included DH727.2 and other antibodies depicted in red shades. **d** DH753 bound the V3 hairpin head-on by virtue of a deep pocket in its paratope. **e** Structures of DH753 with two different V3 peptides were determined in order to scrutinize requirements for interaction. Some conservative mutations are tolerated in the antibody-antigen interface. For instance, mutation of the long Arg308 side chain to the shorter His became a through-interaction mediated by a phosphate ion.

We determined the crystal structure of two of the vaccine-induced antibodies bound to V3 peptides to determine the roles of amino acids identified in the neutralization signature analysis. First, we solved the crystal structure of DH753 in complex with ZAM18 (NNTRKSIRIGPGQAFYATGGIIG), a sequence closely resembling clade A and C virus sequences (Fig. 5a and Supplementary Fig. 5a). Second, we solved DH753 bound to a clade B MN V3 peptide (YNKRKRIHIGPGRAFYTTKNIIG; Supplementary Fig. 5c). The two peptides were chosen because their structures in complex with antibody 3074 have been previously solved^32^. Structures of the complexes were determined to 2.2 and 2.7 Å resolution, respectively (Supplementary Table 2). The DH753:ZAM18 structure showed electron density for residues 305–320 and the MN complex structure showed electron density for residues 306–321. Both DH753 structures showed the V3 loop peptides bound in the same orientation with an RMSD of 0.861Å^2^ when the DH753 Fv regions were aligned (Supplementary Fig. 5c). DH727.2 Fab was crystallized in complex with ZAM18 V3 peptide (gp120_301-325_) to 1.8 Å resolution (Fig. 5b, Supplementary Fig. 5b and Supplementary Table 2). Electron density of the V3 peptide was sufficient to build 14 residues comprising the central segment (residues 305–318) of the peptide (Fig. 5b). The peptide adopted the β-hairpin conformation typical of similar polypeptides representing the V3 crown^32,35–41^.

### Side- and crown-approach modes of V3 peptide recognition

Structural analysis of liganded-DH727 confirmed that DH727.2 had an overlapping epitope with V3 Ab 3074^32^ (Fig. 5c). Also, DH727.2 showed similarity to other V3 peptide antibodies in that its paratope exhibited a defined cleft between the heavy and light chains into which the β-hairpin structure of the V3 crown bound^32,39^ (Fig. 5b and Supplementary Fig. 5b). For DH727.2, this cleft was predominantly formed on one side by HCDR1 and HCDR2 and on the other side by LCDR1 and LCDR3. DH727.2 further resembled other V3 crown antibodies in that it showed an oblique approach to the V3 crown β-hairpin conformation (Fig. 5c). In contrast, DH753 bound the V3 β-hairpin head-on at its apex (the β-turn itself) (Fig. 5a, c). A side approach mode of binding is more frequently observed among V3 antibodies; however, there are other examples of crown approach antibodies as well (Fig. 5c). DH753 showed a rare, exceptionally deep binding pocket on its paratope (Fig. 5d), which has only been observed for one other V3 antibody, 537–10D^39^.

Primary interactions between antibody DH727.2 and antigen were hydrogen (H)-bonds between the terminal amides of the V3 Arg308 side chain with the side chain hydroxyl and backbone carbonyl of Thr97 on the light chain. H-bonds were also observed with the side chain of V3 residue Gln315 and both heavy chain residue Asn59 and light chain residue Thr100. These interactions provided a structural explanation for why Arg308 and Gln315 were found to be a virus sequence signature and were important for peptide binding (Figs. 3b and 4b). Lastly, favorable H-bonding was also observed between the backbone carbonyl of V3 residue Ile309 and the side chain of Arg99 in HCDR3.

In complex with ZAM18 peptide, the binding interface between DH753 and V3 included a dual salt bridge between the side chains of heavy chain residue Asp53 and V3 residue Arg308 (Fig. 5e). Thus, Arg308 was a common contact residue for DH727.2 and DH753. Also, light chain residues Arg53 and His100 interacted with Tyr318 in the V3 peptide. Tyr318 is largely an immutable residue in V3 sequences^32^, which may explain why V3 peptides containing this amino acid bound better to DH753 in the peptide microarray (Fig. 3b). Additonally, contacts between antibody and the V3 polypeptide backbone occurred between the side chain of HCDR3 residue Tyr100B and V3 residue Ile309 as well as heavy chain residue Trp47 with V3 residue Pro313 (in the GPGx arch). In complex with MN peptide, DH753 could not establish the above noted salt bridge between the heavy chain Asp53 and V3 Arg308 side chains since ZAM18 possessed a His at position 308 (Fig. 5e). However, an intermediary phosphate polyatomic ion mediated a polar interaction between the two side chains. This phenomenon was present in both Fab-peptide complexes in the asymmetric unit. The LCDR3 residue Trp91 had van der Waals contacts with Gln315 in the ZAM18 peptide and Arg315 in the MN peptide, explaining why polymorphisms were tolerated at that position (Fig. 5e).

### Molecular modeling of V3 antibodies bound to trimeric Env

**Fig. 6:**
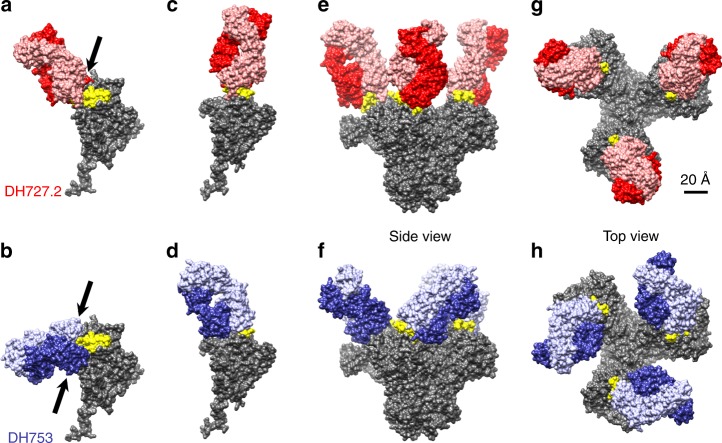
Molecular models predict that DH753 and DH727.2 interact with trimeric HIV-1 Env in the open conformation. **a**–**d** Docking DH727.2 and DH753 onto to a single gp120 from a closed envelope trimer (PDB: 4ZMJ, https://www.rcsb.org/structure/4ZMJ) indicates clashes, marked by arrows where the polypeptide chains overlap (**a**,**b**); whereas docking onto a CD4-induced, open gp120 monomer (PDB: 5VN3 https://www.rcsb.org/structure/5VN3) does not show any clashes (**c**, **d**). **e**–**h** The open trimer can accommodate three copies of either Fab, as shown in the side view (**e**, **f**) and top view (**g**, **h**). Colors are the same as in Figure 5, red/salmon indicate DH727.2, lilac/purple represent DH753, yellow shows the distal end of the V3 loop (residues 301-323), and gray depicts gp120/gp41. The scale is equal in all images

To model potential antibody:Env interactions, we superimposed the Fab:peptide structures onto several Env models by aligning the V3 peptide to the Env, and allowing this alignment to determine the hypothetical orientation of the Fab. When the DH727 structure was aligned onto a single gp120 of a closed envelope trimer there was unrealistic overlap (Fig. 6a), giving 102 clashes between backbone atoms of the Fab heavy chain and the V1V2 domain. DH753 showed 150 backbone clashes that involved the Fab light chain overlapping the V1V2 domain and the heavy chain overlapping the region below the V3 loop (Fig. 6b). These observed clashes between Fab and the V1V2 domain may explain why short V1V2 regions were determined to be a signature of viruses sensitive to V3 antibody neutralization (Fig. 4a). Thus, Fab binding seemed to require opening of the trimer, which would rearrange the V3 loop and/or the V1V2 domain. In agreement with this notion, in the CD4-induced structure of gp120, the V3 loop projects away from the gp120 core and would be freely available to bind DH727.2 or DH753 Fab (Fig. 6c–d). When modeled onto a fully open trimer either Fab would be able to bind freely without clash (Fig. 6e–h).

### V3 neutralizing antibodies lack binding to closed Envs

**Fig. 7:**
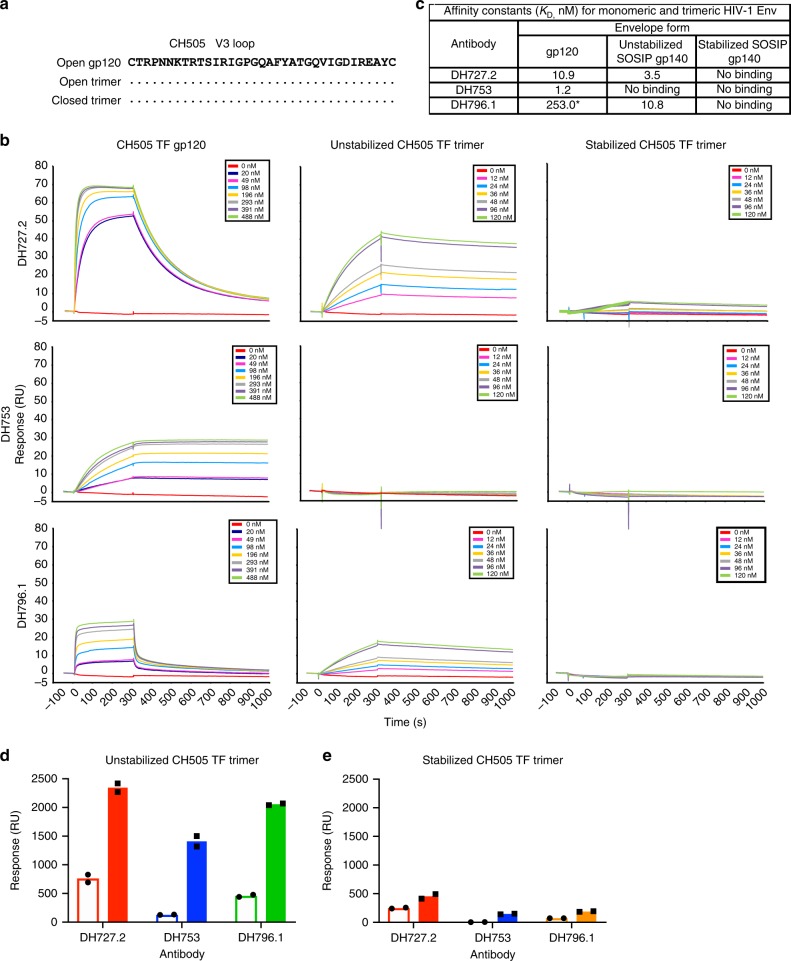
Closed Env conformation limits recognition of the V3 loop by tier 2 neutralizing, vaccine-induced antibodies. **a** CH505 TF gp120 and SOSIP trimer V3 amino acid sequence alignment. Amino acids idenitical to CH505 gp120 are shown as dots. **b** Surface plasmon resonance sensograms of antibody binding to serial dilutions of CH505 TF gp120, unstabilized SOSIP trimer that transitions to the open conformation, and SOSIP trimer stabilized in the closed conformation. Each row shows binding by an individual antibody. Each column shows binding to the specified version of HIV-1 Env. **c** Apparent equilibrium dissociation constants (K_D_) for each antibody binding to the different forms of recombinant Env shown in **b**. Antibodies with binding magnitudes too weak to measure a K_D_ are listed as no binding. **d**, **e** Induction of trimer opening by soluble CD4 augments antibody binding to unstabilized trimers but not conformationally fixed, stabilized trimers. Surface plasmon resonance binding of rhesus monoclonal antibodies DH727.2, DH753, and DH796.1 to CH505 TF Env in the absence (open bar) or presence (filled bar) of soluble CD4. Antibody binding to **d** unstabilized CH505 TF trimer and **e** stabilized CH505 TF trimer is shown. The stabilized Env trimer (DS.SOSIP) contains the 201C-433C disulfide bond that prevents CD4 triggering. Symbols indicate independent measurements, and the bars represent the mean of the duplicate independent experiments. Source data are provided as a Source Data file

Soluble CD4 preincubation experiments have suggested conformational masking of the V3 loop precludes antibody binding to Env^12^. We determined the role of conformational masking in DH727.2, DH753, and DH796.1 binding to recombinant gp120, unstabilized SOSIP.664 gp140, and stabilized DS.SOSIP.664 gp140^13,42,43^. The unstabilized SOSIP trimer is recognized by 19B and 17B and thus sanples the CD4-triggered state of the trimer^43^. In contrast, the CH505 TF DS.SOSIP would be more similar to a pre-CD4 bound Env as it is devoid of binding by CD4-induced antibody 17B^42^. Moreover, the DS.SOSIP possesses a disulfide bond between Cys201C and Cys433 that prevents conformational transitions to the open conformation^13^. Each envelope was derived from the CH505 transmitted/founder virus, therefore, their V3 loop sequences were identical (Fig. 7a). Each antibody bound to the CH505 TF gp120 (Fig. 7b, first column). Presentation of the V3 loop in the context of an Env trimer reduced DH753 binding to below detectable levels. DH796.1 and DH727.2 were able to engage the unstabilized trimer that frequently samples the open conformation, although association with trimer was slower than with the gp120 (Fig. 7b, second column). For DH727.2 and DH796.1 the dissociation from trimeric Env was slower than monomeric gp120 resulting in overall improved binding affinities of 3.5 and 10.8 nM respectively (Fig. 7c). When the trimer was stabilized in the presumably pre-CD4 triggered state^13^ none of the V3 antibodies were able to engage the Env to detectable levels. Thus, conformational fixation of the pre-CD4 bound state of the Env trimer rendered the V3 region inaccessible by the three rhesus macaque antibodies, showing that for these three V3 antibodies, Env conformation dictated antibody binding to CH505 TF Env trimer.

To further explore the Env trimer conformations recognized by DH727.2, DH753, and DH796.1 we triggered envelope into the open state by the addition of CD4 and measured antibody binding to the envelope^4,44^. In contrast to binding affinity experiments, saturating concentrations of antibody were coated on SPR sensor chips to allow avidity to contribute to binding. Inducing the unstabilized into the CD4-induced open state increased binding by the V3 antibodies (Fig. 7d). The increase in binding suggested that the unstabilized trimer possessed some molecules that were not in the fully open conformation at equilibrium. Binding to the stabilized, conformationally-fixed trimer was low and the addition of CD4 did not improve binding to this Env (Fig. 7e)^13^. Taken together, the antibodies bound optimally to Env trimers that were capable of sampling multiple states, including the open state, but poorly recognized trimers that did not transition from the closed state.

### Ontogeny of tier 2 neutralizing V3 antibodies

We investigated the ontogeny of the V3 antibodies from vaccination by isolating two additional members of the DH727 lineage and 5 additional DH796 members (Supplementary Table 1). A maximum likelihood phylogenetic tree was constructed to infer the unmutated common ancestor (UCA) and intermediate antibodies of the DH727 and DH796 clonal lineages (Supplementary Fig. 6a). Of the three antibody lineages only the DH727 UCA was capable of binding HIV-1 envelope (Supplementary Fig. 6b). The DH727 UCA bound to the vaccine immunogen CH505 TF gp120 and V3 peptides from clade C consensus (Con C) and CON-S viruses. For the DH727 and DH796 antibody lineages, the somatically mutated early intermediate antibodies in the lineages bound with higher titers than the UCA (Supplementary Fig. 6b). The DH727 UCA neutralized CNE8 (IC50 = 0.43 μg mL^−1^) as did all other members of the DH727 lineage (Supplementary Fig. 6c). The minimally mutated lineage members also showed weak neutralization of tier 2 virus 25710-2.43 (IC50 range = 6–30 μg mL^−1^) (Supplementary Table 1; Supplementary Fig. 6c). The DH753 UCA and DH796 UCA did not neutralize any of the 6 viruses assessed, but early DH796 intermediate antibodies neutralized 3 of 6 viruses examined (Supplementary Fig. 6c). Therefore, neutralization activity arose with only little somatic mutation.

## Discussion

Here we demonstrated that Env V3 loop antibodies induced by vaccination can neutralize HIV-1 primary isolates categorized as tier 2, difficult-to-neutralize viruses. Studies have shown that V3 region antibodies could neutralize select primary isolates, but lacked detectable breadth^9,31,45–47^. In this study we performed a comprehensive assessment of the neutralization breadth of V3 region antibodies on large standardized panels of 292 HIV-1 viruses to definitively show the neutralization potential of tier 2 virus-neutralizing V3 region-specific antibodies. These large virus panels are the gold standard for determining HIV-1 neutralization breadth, and are used to signal the presence of bnAbs or their precursors across vaccine trials and infected human samples^19,26,27^. However, we determined that weak neutralization of viruses within these standardized panels can be due to V3 antibodies—not antibodies against the known broadly neutralizing epitopes on tier 2 HIV-1 Envs with closed conformations.

Recent vaccination studies have reported weak or moderate neutralization of tier 2 HIV-1 strains^45,47,48^. Comparing a recent report of inducing tier 2 neutralizing antibodies in macaques to the isolates neutralized by the V3 antibodies in this study, 3 of 4 viruses were sensitive to the V3 antibodies^29^. Thus, when low titer neutralization is observed in polyclonal sera care must be taken to rule out V3-specific neutralizing antibodies as the mediators of the tier 2 or primary isolate neutralization. Also, it should be noted that not all HIV-1 V3 antibodies possess the same neutralization breadth, and the breadth can be highly clade dependent (Fig. 2). In our combined viral panels, DH796.1 was more potent and broader than the other vaccine-induced V3 antibodies as well as the best-in-class V3 antibodies from human infection 3074, and 447-52D (Supplementary Data 1). Therefore, a particular isolate may be resistant to several known V3 monoclonal antibodies, but it should not be assumed that infection-induced or vaccine-elicited polyclonal sera do not contain V3 antibodies capable of neutralizing those viruses. The complexities of interpreting the low neutralization titers from sera or plasma highlight the importance of identifying the monoclonal antibodies that mediate the neutralization activity, or at the very least performing definitive plasma neutralization mapping experiments.

HIV-1 neutralization by these newly isolated V3 antibodies often did not reach 100 percent. This incomplete neutralization has been observed in the past for glycan-dependent antibodies^28^, which may be due to differences among Env glycosylation profiles. The underlying mechanisms for incomplete neutralization by V3 region antibodies are speculative at present, but may be related to Env structural dynamics^21,49^ or glycosylation changes^15^. Amino acid sequence is clearly important as our signature analysis showed that certain Env sequences were preferred by each V3 antibody, and these preferences contributed to clade specificity for each antibody. Additionally, the Env structure was important for recognition as well. In surface plasmon resonance experiments, Env trimer conformations antigenically resembling the CD4-induced state were bound by V3 antibodies with high magnitude, whereas soluble trimers stabilized in a non-CD4-induced or closed conformation eliminated binding. SmFRET analyses have suggested that Env conformation on virions can be present in multiple states^21^. Thus, it is possible that a subset of these states, but not all of these states, can be targeted by V3 antibodies. The changes in Env conformation may be subtle, and only affect the V3 region since V3 can change positions without disrupting the adjacent V1V2 region^15^. Previous studies have suggested Env breathing or V3 flickering as instances where HIV-1 Env conformation undergoes transient changes^15,50^. Transient exposure of V3 on a subset of virions would be consistent with the incomplete neutralization exhibited since only a portion of virions would have Envs in a conformation antigenic for V3 antibodies at any one time.

The protective capacity of V3-specific antibodies after vaccination is controversial. While V3 antibodies with effector functions may contribute to protection^51^, neutralization by V3-specific antibodies has not been shown to protect against infection by tier 2 SHIV challenges after vaccination^46^. This lack of evidence of protection from infection may be because of the lack of complete neutralization of primary, difficult-to-neutralize viruses. In contrast to horizontal transmission, V3 antibodies were identified as a correlate of decreased transmission risk in mother-to-child-vertical transmission of HIV-1^52^. In a cohort of women, who were not ART-treated, V3-specific IgG was higher in non-transmitting women than transmitting women^52^. The V3 antibodies from the Women and Infant Transmission Study (WITS) were capable of neutralizing autologous primary isolates, but not heterologous primary isolates^52^. Such autologous V3 region neutralizing antibodies could serve to cull viruses so that only V3-resistant viruses are present to be transmitted. The protective V3 antibodies seemed to bind to the C-terminal portion of the V3 region^53^, which would be similar to the binding mode of DH753. Either Env vaccination of or passive transfer of DH796.1 into pregnant or breast-feeding macaques that are infected with simian-human immunodeficiency virus could determine whether V3 antibodies could reduce vertical transmission risk providing a biological significance to these antibodies.

In summary, we demonstrate the heterogeneity of V3 loop expression on tier 2 difficult-to-neutralize HIV-1 strains in current standard panels of HIV-1 viruses, and demonstrate caution should be exercised in the interpretation of vaccine-induced neutralization of such strains.

## Methods

### Animals and immunizations

Rhesus macaques were housed and treated in AAALAC-accredited institutions. The study protocol and all veterinarian procedures were approved by the Duke University IACUC and were performed based on standard operating procedures. Rhesus macaques were immunized with CH505 gp120 monomers. The CON-S gp140 envelopes were uncleaved, unstabilized gp140 lacking the furin cleavage site, fusion peptide, and gp41 immunodominant region (CFI). The VRC immunogens were unmodified envelope gp160s.

### Crystallography

Fabs were expressed in transiently-transfected Expi293 cells (Invitrogen, Cat No. A14527)^54^. The Fab was purified by KappaSelect affinity chromatography. The Fab was eluted off of the KappaSelect resin with a glycine pH2.4 buffer and the pH was subsequently neutralized by the addition of Tris pH8.0. The Fab was then further purified via size exclusion chromatography. Peak protein-containing fractions were concentrated, buffer exchanged to ddH2O, and brought to 15.0 mg ml^−1^. Fabs were mixed with V3 peptides corresponding to gp120_301–325_ in a 1:3 molar ratio. The V3 peptides were replicated from previously published V3 crown antibody structures^32^. The complexes were tested against commercially available screens (Qiagen, Molecular Dimensions) in SBS format sitting drop plates via automation (Douglas Instruments Ltd) with 60μl reagent reservoirs and drops composed of 0.2μl protein with 0.2μl reservoir. Crystals for DH727.2 with ZAM18 peptide were observed over a reservoir of 0.1 M Tris pH 8.5, 20% PEG 6,000. Crystals for DH753 with ZAM18 peptide were observed against a reservoir of 0.2 M sodium sulfate decahydrate, 20% PEG 3350. Crystals for DH753 with MN peptide were observed against a reservoir of 0.2 M ammonium dihydrogen phosphate, 20% PEG 3350. All crystals were briefly soaked in reservoir supplemented with ~20% ethylene glycol then flash-frozen in liquid nitrogen.

Data were collected at Southeast Regional Collaborative Access Team (SER-CAT) 22-ID (or 22-BM) beamline at the Advanced Photon Source, Argonne National Laboratory. Diffraction data for all crystals were collected at SER-CAT with an incident beam of 1 Å in wavelength. The DH727.1 + ZAM18 structure showed 96.8% of amino acid residues in favored areas of the Ramachandran plot with none in outlying regions. The DH753 + ZAM18 structure similarly showed 97.0% of residues in favored regions with no outliers. The DH753 + MN structure had 93.1% of residues in favored regions with just 0.8% in outlying regions. Data were reduced in HKL-2000^55^. The DH727.2-ZAM18 peptide structure was phased by molecular replacement in PHENIX^56^ using as the search model the Fab fragment of an antibody to Madcam-1 Dld2 as selected by high sequence identity^57^. Likewise, the DH753-ZAM18 structure was phased using a composite search model generated from the Fab fragment of the HIV antibody 10E8 germline heavy chain^58^ with the HIV antibody DH501 light chain^24^. The DH753-MN structure was in turned using the DH753-ZAM18 structure as the search model. Rebuilding and real-space refinements were performed in Coot^59^ with reciprocal space refinements in PHENIX^60^ and validations in MolProbity^61^.

### Molecular modeling of antibody bound to HIV-1 trimer

All modeling was done using UCSF Chimera^62^. To model the Fab interactions with the closed gp120, we used chain G from a BG505 SOSIP structure (PDB: 4ZMJ[10.2210/pdb4ZMJ/pdb])^13^, and docked the Fab-peptide structures reported here onto the closed gp120, using only the peptide fragment of the Fab structure for alignment onto the gp120. Clashes between the gp120 and the Fab were calculated with Chimera’s Find Clashes/Contacts function, using the default clash settings and specifying only backbone atoms. For the open monomer and trimer, we built homology models for the CH505 TF SOSIP sequence using the open BG505 structure (PDB: 5VN3[10.2210/pdb5VN3/pdb])^44^ as a template for SWISS-MODEL^63^, and docked the Fabs as above.

### HIV-1 Env peptide array

The HIV-1 peptide libraries contain overlapping HIV-1 peptides covering full-length gp120 of 5 consensus viruses from group M and clades A, B, C, and D^33^. Array slides were provided by JPT Peptide Technologies GmbH (Germany) by printing a library of peptides onto epoxy glass slides (PolyAn GmbH, Germany). The library contains overlapping peptides (15-mers overlapping by 12) covering 5 full-length gp160 consensus sequences (clade A, B, C, D, and group M). V3 peptide binding breadth was analyzed for a library of V3 peptides (15-mers overlapping by 12) for 7 consensus sequences (clade A, B, C, D, CRF1, and CRF2, and group M) and 6 vaccine strains (MN, A244, TH023, TV-1, ZM651, 1086 C). Three identical subarrays were blocked for 1 h, followed by a 2-h incubation with monoclonal antibody, and a subsequent 45-min incubation with anti-monkey IgG conjugated with AF647 (Jackson ImmunoResearch, PA). Array slides were scanned at a wavelength of 635 nm using an InnoScan 710 scanner (InnopSys, Denmark) and images were analyzed using Magpix V8.1.1.

### Antigen-specific single B cell sorting

Cryopreserved PBMC were washed and counted^64^. The PBMC was stained with NK, T, and B cell surface markers and fluorophore-labeled envelope protein for 1 h at 4 °C. Antibodies used for staining were CD20 FITC clone L27 (BD Biosciences Cat No. 347673), CD3 PerCP Cy5.5 clone SP34-2 (BD Biosciences Cat No. 552852), IgD PE polyclonal (Southern Biotech Cat No. 2030-09), CD8 PE Texas Red clone 3B5 (Invitrogen Cat No. MHCD0817), IgM PE Cy5 clone G20-127 (BD Biosciences Cat No. 551079), CD16 PE Cy7 clone 3G8 (BD Biosciences Cat No. 557744), Live / Dead Aqua (Invitrogen Cat No. L34957), CD14 BV570 clone M5E2 (BioLegend Cat No. 301832), and CD27 APC Cy7 clone O323 (BioLegend Cat No. 302816). Envelope reactive, live, IgD- single B cells were sorted into individual wells of a PCR plate that contained lysis buffer. Plates were frozen on dry ice and ethanol and stored at −80 °C until PCR amplification of immunoglobulin genes.

### Rhesus immunoglobulin RT-PCR

Immunoglobulin genes from a single B cell were reverse transcribed with Superscript III (ThermoFisher) and gene-specific reverse primers that anneal in the constant regions of each isotype (Supplementary Table 3)^25,64^. The complementary DNA was used as template for two rounds of nested PCR for heavy and light chain gene amplification. Positive PCR amplification of immunoglobulin genes was identified by agarose gel electrophoresis. Positive PCR reactions were purified using the PCR clean-up kit (Qiagen). The sense and antisense strands of the purified PCR amplicon were sequenced with 4 μM of forward and reverse primers. Contigs of the PCR amplicon sequence were made, and each gene segment was inferred with the rhesus library in Clonanalyst^64,65^. The unmutated common ancestor (UCA) antibodies were inferred using the rhesus library in Clonanalyst. A second aliquot of the purified PCR amplicon was used for overlapping PCR to generate a linear expression cassette. The expression cassette was transfected into 293 T cells (ATCC, Cat No. CRL-11268) with Efectene (Qiagen). Cell culture media containing recombinant antibodies were tested for binding to HIV-1 envelope. The genes of selected heavy and light chains were synthesized (GenScript). Plasmids were prepared for transient transfection using the Megaprep plasmid plus kit (Qiagen).

### Monoclonal antibody competition ELISAs

Nuncsorp plates were coated with HIV-1 envelope, washed and blocked with Superblock^24^. After blocking was complete, non-biotinylated monoclonal antibodies were serially diluted in SuperBlock starting at 100 μg mL^−1^ and incubated in triplicate wells for 90 min. To determine relative binding no antibody was added to a group of wells scattered throughout the plate. After 90 min the non-biotinylated antibody was washed away and biotinylated monoclonal antibodies or soluble CD4 was incubated in the wells for 1 h at sub-saturating concentrations. The binding of CD4 was detected with biotinylated anti-CD4 antibody OKT4. As a positive control, the same antibody was used to block itself. For CD4 blocking assays the CD4 binding site antibody CH106 was used as positive control antibody. As a negative control an anti-influenza antibody CH65 was added to the Env prior to addition of the biotinylated monoclonal antibodies or soluble CD4. Each well was washed, and binding of biotinylated antibodies was determined with a 1:30000 dilution of horseradish peroxidase (HRP)-conjugated streptavidin. HRP was detected with tetramethylbenzidine and stopped with 1% HCl. The absorbance at 450 nm of each well was read with a Spectramax plate reader (Molecular Devices). Binding of the biotinylated monoclonal antibody to HIV-1 envelope in the absence of competing antibody was compared to in the presence of competing antibody to calculate percent inhibition of binding. Based on historical negative controls, assays were considered valid if the positive control antibodies blocked greater than 20% of the biotinylated antibody binding.

In vitro HIV-1 neutralization. Antibody-mediated HIV-1 neutralization was measured using Tat-regulated luciferase (Luc) reporter gene expression to quantify reductions in virus replication in TZM-bl cells^66^. TZM-bl cells were obtained from the NIH AIDS Research and Reference Reagent Program, as contributed by John Kappes and Xiaoyun Wu. The monoclonal antibody was pre-incubated with virus (~150,000 relative light unit equivalents) for 1 h at 37 °C, and TZM-bl cells were subsequently added. After 48 h cells were lysed and Luc activity determined using a microtiter plate luminometer and BriteLite Plus Reagent (Perkin Elmer). Neutralization titers are the inhibitory concentration at which relative luminescence units (RLU) were reduced by 50% or 80% compared to RLU in virus control wells after subtraction of background RLU in cell control wells (IC50 and IC80 respectively).

Recombinant antibody production. Expi293 cells (Invitrogen, Cat No. A14527) were cultured in Expi293 media at less than 5 × 10^6^ cells mL^−1^. On the day of transfection cells were diluted to a final volume of 0.5 L at a concentration of 2.5 × 10^6^ cells mL^−1^ in Expi293 media. Expi293 cells were co-transfected with 400 μg of heavy chain plasmid and light chain plasmid using Expifectamine. Five days after transfection cell culture media was cleared of cells by centrifugation and 0.8 μM filtration. The cell-free supernatant containing IgG1 was incubated with protein A resin (ThermoFisher) overnight at 4 °C. The protein A resin was centrifuged and cell culture supernatant was removed. The resin was washed with 25 mL of PBS with 340 mM NaCl. Thirty mL of 10 mM glycine pH 2.4, 150 mM NaCl were used to elute the antibody off of the affinity resin. The pH of the eluted antibody solution was increased to neutral pH by adding 1 M Tris pH8.0. The neutral pH eluate was buffer exchanged into PBS with successive rounds of centrifugation, filtered, and stored at −80 °C.

Recombinant SOSIP Env production. Freestyle 293 (Invitrogen, Cat No. R79007) cells were cultured in Freestyle 293 media below 3 × 10^6^ cells mL^−1^. On the day of transfection, cells were diluted to 1.25 × 10^6^ cells mL^−1^ with fresh media and 1 L of cells was transfected with 293Fectin (Life Technologies) and 650 and 150 μg of SOSIP and furin expressing plasmid DNA respectively. Cells were cultured for 6 days at 37 °C and 8% CO_2_ in an humidified incubator. At the end of the transfection, cell cultures were centrifuged for 30 min at 3500 rpm and 0.8μm filtered. The cell-free supernatant was concentrated to less than 100 mL with a single-use tangential flow filtration cassettes and 0.8 μm filtered again. Trimeric Env protein was purified with PGT145 affinity chromatography. One hundred mg of PGT145 IgG1 antibody was conjugated to 10 mL of CnBr-activated sepharose FastFlow resin (GE Healthcare). Coupled resin was packed into Tricorn column (GE Healthcare), and stored in PBS supplemented with 0.05% sodium azide. Cell-free supernatant was applied to the column at 2 mL min^−1^ using an AKTA Pure (GE Healthcare), washed, and protein was eluted off of the column with 3 M MgCl_2_. The eluate was immediately diluted in 10 mM Tris pH8, 0.2 μm filtered, and concentrated down to 2 mL for size exclusion chromatography. Size exclusion chromatography was performed with a Superose6 16/600 column in 10 mM Tris pH8, 500 mM NaCl. Fractions containing trimeric HIV-1 Env protein were pooled together, sterile-filtered, snap frozen, and stored at −80 °C.

Recombinant Env gp120 production. Recombinant gp120 was expressed in Freestyle 293 cells (Invitrogen, Cat No. R79007)^24^. Cells were transfected with PEI:DNA complexes, and cultured for 5 days. Recombinant protein was purified with *Galanthus nivalis* lectin-agarose (Vector Laboratories), buffer exchanged into phosphate buffered saline and stored at −80 °C.

Phylogenetic Trees. Maximum likelihood trees were generated using PhyML^67^ with the HIVb model^68^ (https://www.hiv.lanl.gov/content/sequence/PHYML/interface.html), and represented using Rainbow Tree at the Los Alamos database (https://www.hiv.lanl.gov/content/sequence/RAINBOWTREE/rainbowtree.html).

### Neutralization signature analysis

We performed a phylogenetically corrected analysis with a liberal threshold of *q* = 0.3 for an inclusive sweep of the full protein alignment. We next used a simple signature analysis, with no phylogenetic correction, to identify potential signatures associated with neutralization sensitivity within the V3 loop region, including the PNGS sites that bound it on either side at N295 and N332. Because these sites are near the antibody contact regions, they are good candidates for direct involvement in antibody interactions, and may be responsible for the antigenic phenotype of the Env as well as for the clade specificity of V3 antibodies. But without the phylogenetic correction it is also possible that these signatures may be carried along with the site or sites that more directly determine the positive/negative sensitivity phenotype. For example, Arg315 is required for 447-52D neutralizing activity and it is highly enriched in the B clade relative to other clades. Other amino acids that are specifically enriched in the B clade may also be associated with 447-52D, that are not directly impacting the phenotype but are genetically linked to Arg315. The LOGOS of the signature sites were created using the Los Alamos database Analyze Align tool (https://www.hiv.lanl.gov/content/sequence/ANALYZEALIGN/analyze_align.html).

### Direct ELISA

In total 2 μgmL^−1^ of protein in sodium bicarbonate buffer was incubated in sealed Nunc-absorp (ThermoFisher) plates overnight at 4 °C^24^. Unbound protein was washed away and the plates were blocked with SuperBlock for 1 h. Serially dilution of monoclonal antibodies were added to the plate for 90 min. Binding antibodies were detected with 1:30,000 dilution of HRP labeled anti-IgG Fc antibodies (Southern Biotech, SB108a, Cat no. 4700-05). HRP was detected with 3,3′,5,5′-Tetramethylbenzidine. Binding titers were analyzed as area-under-curve of the log-transformed concentrations.

### Surface plasmon resonance (SPR)

SPR experiments were performed on a BIACore T200. For kinetics measurements approximately 50 RU of each antibody was captured on an anti-human IgFc immobilized Series S CM5 sensor chip (GE Healthcare). Serial dilutions of HIV-1 Env was flowed over the chip in HEPES buffered saline. The Env proteins were CH505 transmitted founder gp120, unstabilized chimeric 6 R.SOSIP.664 gp140, and stabilized chimeric 6R.DS.SOSIP.664 gp140^43^. The stabilized chimeric SOSIP contains I201C and A433C mutations to create a stabilizing disulfide bond^13,42^. Envelope concentrations were empirically determined that gave 50–100 RU. Based on these initial range-finding experiments 8 concentrations were selected for kinetic value determination. Each concentration of Env was flowed over each immobilized antibody for 120 s and dissociation was measured for 600 s. In between injections of each Env concentration, the surface was regenerated by injecting glycine pH1.5 for 30 s. Binding rate constants (ka, kd) were measured following global curve fitting to a Langmuir model. Curve fitting analysis was performed with BiaEvaluation software (GE Healthcare) using a 1:1 Langmuir model, or a heterogenous binding model when appropriate to derive rate (ka, kd) and apparent equilibrium dissociation constants (K_D_).

For CD4 triggereing analysis, ~2000 RU of antibody was captured on Series S CM5 sensor chip (GE Healthcare). CH505 transmitted/founder unstabilized chimeric 6 R.SOSIP.664 gp140 or stabilized chimeric 6 R.DS.SOSIP.664 gp140^43^ was incubated with soluble 2-domain CD4. As a control the same Envs were not treated with soluble CD4. CD4 bound Env and free Env were flowed over antibody to assess differences in binding.

### Statistical methods

R implementations of (www.r-project.org/) the following statistical tests were used as noted in the text. A Fisher’s test with a Bonferonni correction for multiple tests was used to compare counts of V2 loop characteristics divided about the median among viruses that were positive and negative for antibody neutralization. Hypervariable region characteristics were defined^69^, and implemented using the hypervariable tool at the Los Alamos HIV website: https://www.hiv.lanl.gov/content/sequence/VAR_REG_CHAR/index.html. The hypervariable region of V2 was defined as the region between HXB2 positions I184 a Y191; this region often mutates by insertion and deletion, which manifests as extreme length diversity. The Kolomogov-Smirnov test was used to compare cumulative distributions of breadth-potency. We used q-values to address multiple tests in the signature analysis^70^.

## Supplementary information

Supplementary Information

Description of Additional Supplementary Files

Supplementary Data 1

Supplementary Data 2

Source Data

## Data Availability

The signature method, which identifies amino acids in positions of a sequence alignment that are associated with a phenotype characterization of the sequences (such as a measure of neutralization sensitivity) with the option of using a phylogenetic correction, was first described by Bhattacharya and colleagues^71^. The code was further developed, and interface for the code called GenSig, made available at the HIV database at Los Alamos National Laboratory (https://www.hiv.lanl.gov/content/sequence/GENETICSIGNATURES/gs.html) in conjunction with the publication by Bricault and colleagues^69^.

## Data availability

The authors declare that the data supporting the findings of this study are available within the main and supplemental figures. Data underlying Figs. 1, 2, 3, 4, and 7 and Supplementary Figs. 2, 3, 4, and 6 are provided as Source Data File. All other data is available from the corresponding author upon reasonable request. Any raw flow cytometric data is available upon request. Coordinates and structure factors have been deposited in the Protein Data Bank under accession codes 6MNQ (DH727.2 + ZAM18 V3 peptide), 6MNR (DH753 + ZAM18 V3 peptide), and 6MNS (DH753 + MN V3 peptide).
