# Supplementary material for: Difficult-to-neutralize global HIV-1 isolates are neutralized by antibodies targeting open envelope conformations
Source: Nat Commun. 2019 Jul 1;10:2898. doi: 10.1038/s41467-019-10899-2 (PMC6602974; doi:10.1038/s41467-019-10899-2)
Supplement: Supplementary file 1 — Supplementary Information [file 41467_2019_10899_MOESM1_ESM.pdf]

Supplementary Information for Difficult-to-neutralize global HIV-1 isolates are neutralized by antibodies targeting open envelope conformations

Han et al.

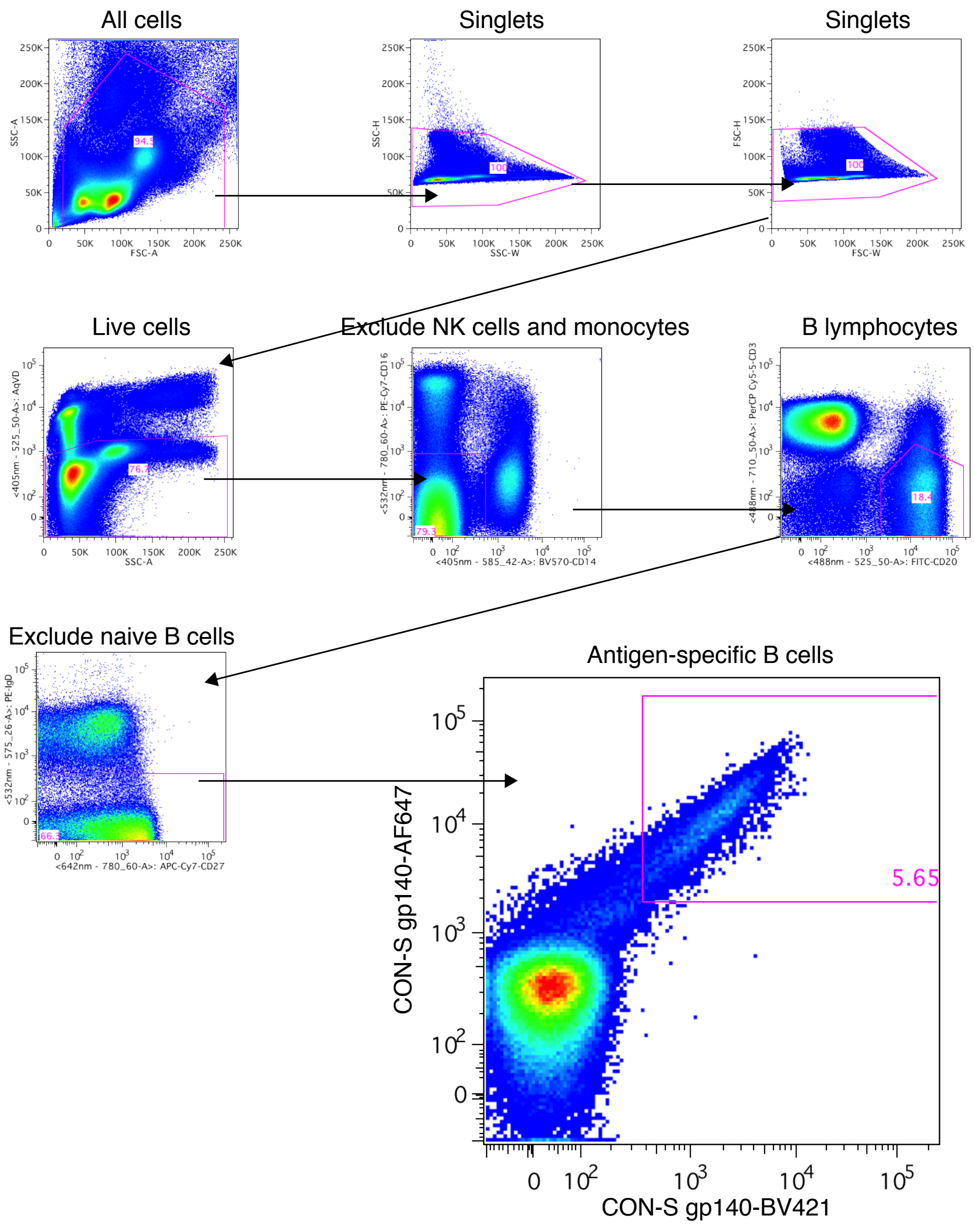

Supplementary Figure 1. Fluorescence-activated cell sorting of envelope-specific B cells. The gating strategy to identify single B cells specific for HIV-1 envelope is shown for L983 as representative for all three B cell sorts performed. The antigen-specific FACS plot for each macaque is shown in Figure 1.

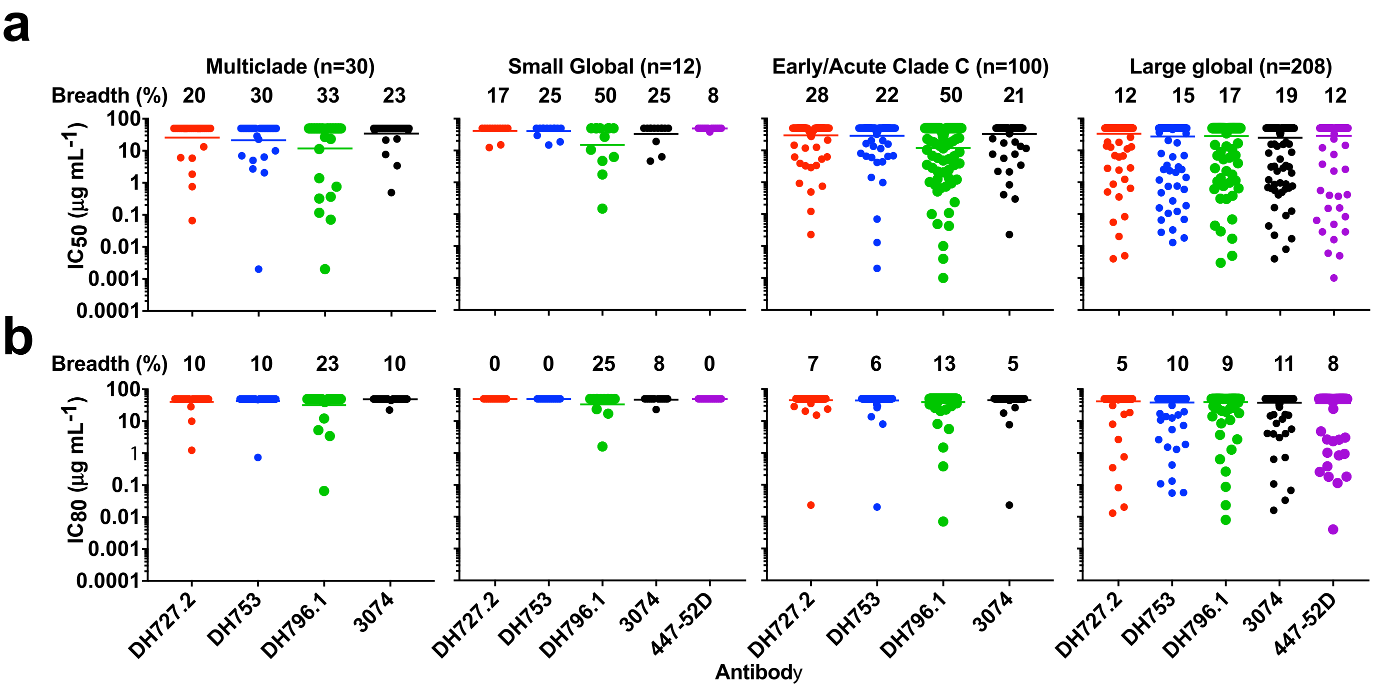

c

GMT of multiclade virus panel / GMT of small global panel

Antibody	DH727.2	DH753	DH796.1	3074	447-52D	DH727.2	DH753	DH796.1	3074	447-52D
IC_{50} (µg mL^{-1})	1.9	3.0	0.7	10.3	nt	13.7	20.2	6.8	8.3	38.4
IC_{80} (µg mL^{-1})	7.1	11.9	7.3	36.8	nt	50.0	50.0	8.6	23.7	50.0

GMT of early/acute clade C virus panel / GMT of large global panel

Antibody	DH727.2	DH753	DH796.1	3074	447-52D	DH727.2	DH753	DH796.1	3074	447-52D
IC_{50} (µg mL^{-1})	7.9	4.0	2.8	6.3	nt	2.8	1.2	2.2	1.6	0.4
IC_{80} (µg mL^{-1})	9.7	6.2	7.1	5.1	nt	1.4	3.0	3.3	3.4	0.9

Supplementary Figure 2. Vaccine-induced antibody neutralization of HIV-1 tier 2 viruses categorized into their standard virus panels.
(a,b) Macaque vaccine-induced antibodies DH727.2, DH753, and DH796.1 *in vitro* neutralization of HIV-1 infection of TZM-bl cells. 3074 and 447-52D are human HIV-1 antibodies from infection that were included for comparison. Neutralization was assessed against four standard panels of viruses. Neutralization titers in µg mL^{-1} are shown as the concentration of antibody that inhibits (**a**) 50% (IC50) or (**b**) 80% (IC80) of virus replication. The neutralization breadth is shown above each column as the percentage of viruses neutralized by 50% or 80%. Each symbol represents an individual virus. The horizontal bar represents the geometric mean of the IC50 or IC80 for all viruses.
(c) The geometric mean titer (GMT) of only the neutralized viruses in each panel. Source data are provided as a Source Data file.

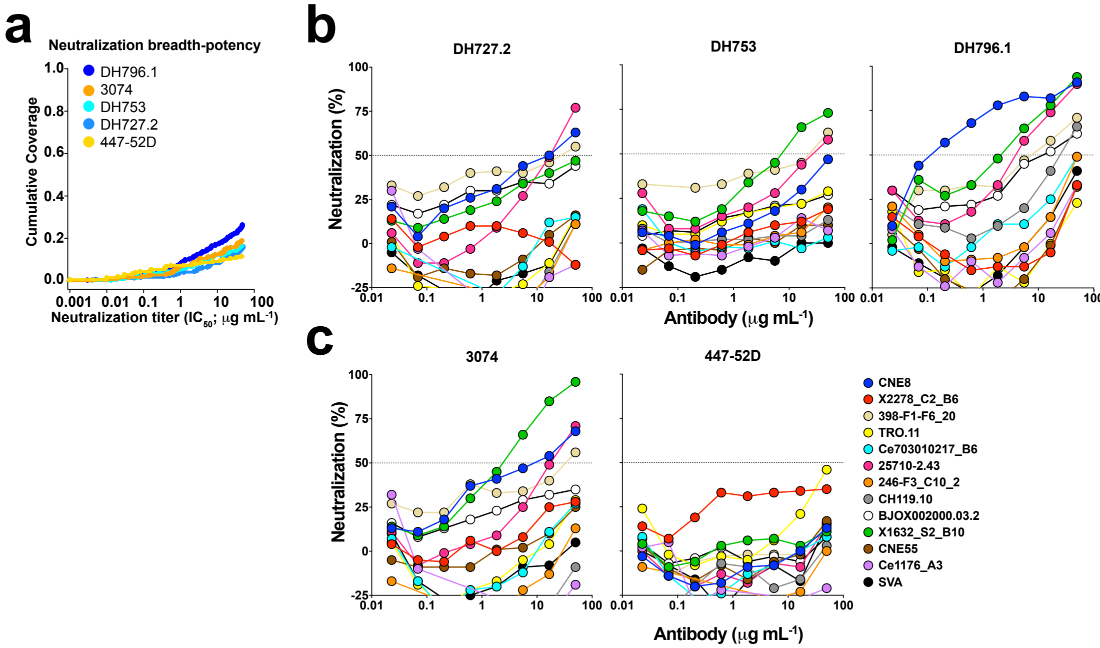

d

Neutralization titer IC50 (µg mL^{-1})				
**Virus**	**DH727.2**	**DH753**	**DH796.1**	**CH01-31**
TT31P.2F10.2792	>25	>25	>25	**0.1**
1051-12.C22.3325	>25	>25	>25	**0.52**
1051-12.TD12.3291	>25	>25	>25	**0.1**
9021-14.B2.4571	>25	>25	>25	**0.2**
700010040.C9.4520	>25	>25	>25	**2**
1058-11.B11.1550	>25	>25	>25	**0.06**
1056-10.TA11.1826	>25	>25	>25	**0.96**
SC05.8C11.2344	>25	>25	>25	**0.65**
PRB926-04.A9.4237	>25	>25	>25	**0.22**
700010058.A4.4375	>25	>25	>25	**0.07**
PRB958-06.TB1.4305	>25	>25	>25	**1.4**
SC45.4B5.2631	>25	>25	>25	**0.26**
1006-11.C3.1601	>25	>25	>25	**0.34**
1059-09.A4.1460	>25	>25	>25	**11**
6240-08.TA5.4622	>25	>25	>25	**6**
PRB931-06.TC3.4930	>25	>25	>25	**0.14**
6244-13.B5.4576	>25	>25	>25	**0.92**
1054-07.TC4.1499	>25	>25	>25	>25
1012-11.TC21.3257	>25	>25	>25	**0.28**
62357-14.D3.4589	>25	>25	>25	>25
63358-04.P3.4013	>25	>25	>25	**3.2**
SVA-MLV	>25	>25	>25	>25

Supplementary Figure 3. Neutralization breadth and potency against diverse global HIV-1 isolates and transmitted/founder viruses.

(a) The breadth-potency is shown as cumulative distributions of the IC50 results for the V3 antibodies based on the 292 viruses in the combined panels (except for 447-52D, which was based on the 208 virus global panel). DH796.1 ranked highest, although the cumulative distribution was not significantly different than 3074 (Kolmogorov-Smirnov $p = 0.38$, $n = 208$ or 292 viruses), and 3074 ranks higher outside of clade C. See the trees in Fig. 2 and the complete dataset in Supplementary Data File 1.

(b,c) Neutralization of 12 global HIV-1 isolates[1] infection of TZM-bl cells by (**b**) macaque vaccine-induced antibodies and (**c**) human antibodies from natural infection. Viruses in this panel with sensitivity to DH727.2, DH753, DH796.1, and macaque BG505 immune sera are shown in Fig. 1h.

(d) DH727.2, DH753, and DH796.1 lack neutralization of selected clade B transmitted/founder HIV-1 isolates. Values are the concentration of antibody in µg mL^{-1} that inhibits 50% of virus replication in TZM-bl cells (IC50). As a positive control for HIV-1 neutralization a mixture of bnAbs CH01 and CH31 was used starting at 25 µg mL^{-1}. Values above 25 µg mL^{-1} are considered negative for neutralization. Neutralization titers are color-coded as follows: white>25; yellow 24.9-5, light orange 4.9-0.5, orange 0.49-0.05, red <0.05. Murine leukemia virus (SVA-MLV) was used as a negative control virus. Source data are provided as a Source Data file.

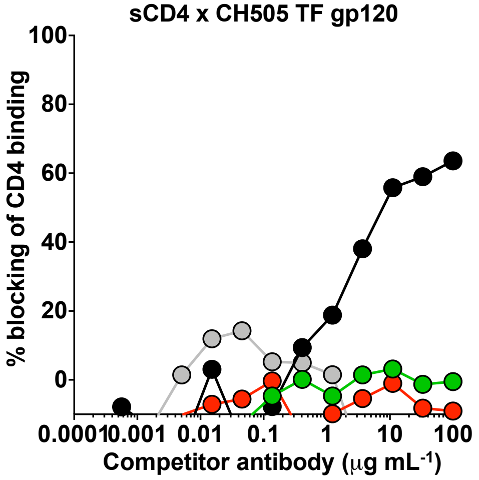

Supplementary Figure 4. Rhesus antibodies do not block CD4 engagement of Env gp120. Soluble CD4 binding to CH505 TF gp120 in the presence of increasing concentrations of rhesus monoclonal V3 antibodies DH727.2 (red), DH753 (blue), DH796.1 (green). Blocking greater than 20% is considered positive. CH106 was used as a positive control (black) and anti-influenza antibody CH65 (gray) was used as a negative control. Source data are provided as a Source Data file.

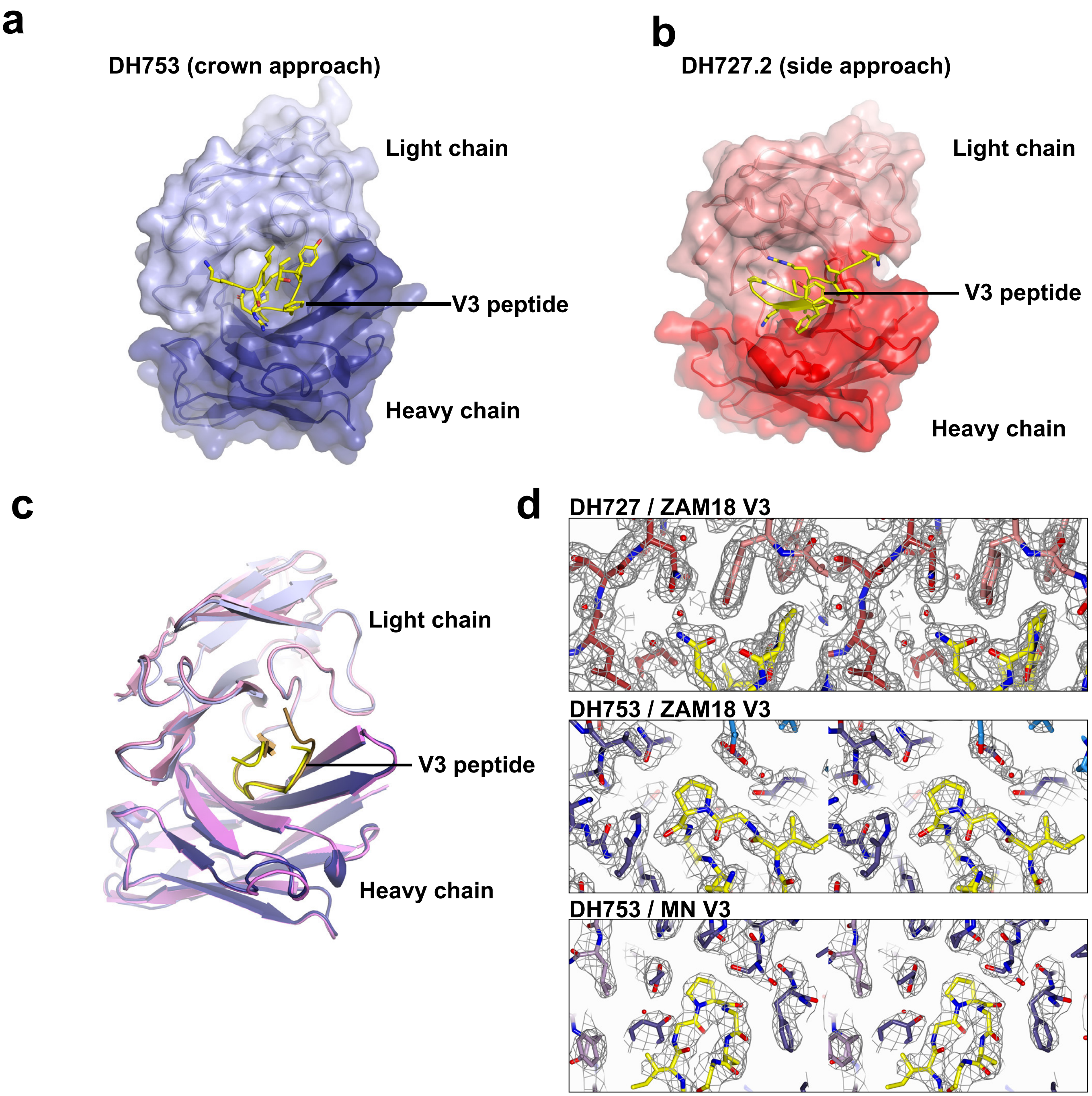

Supplementary Figure 5. V3 peptide side chain interactions with DH753 and DH727.2 Fabs.
(a) The crystal structure of DH753 is shown with the L chain rendered in lilac, the FabH chain in dark purple bound to ZAM18 V3 hairpin peptide in yellow.
(b) The crystal structure of DH727.2 bound to ZAM18 V3 peptide. The Fab is shown in a similar orientation as in **a** with the L chain in salmon, the FabH chain in red.
(c) Superposition of DH753 Fab bound to ZAM18 (yellow) and MN (orange) V3 peptides.
(d) Depicted are stereo images of the 2Fo-Fc representative electron density maps at 1.0 sigma contour levels. The figures are centered on the DH727 interface with ZAM18 V3 peptide (specifically at Gln315) and the DH753 interface with ZAM18 and MN peptides (at Pro313), respectively at a 1.0 sigma contour level.

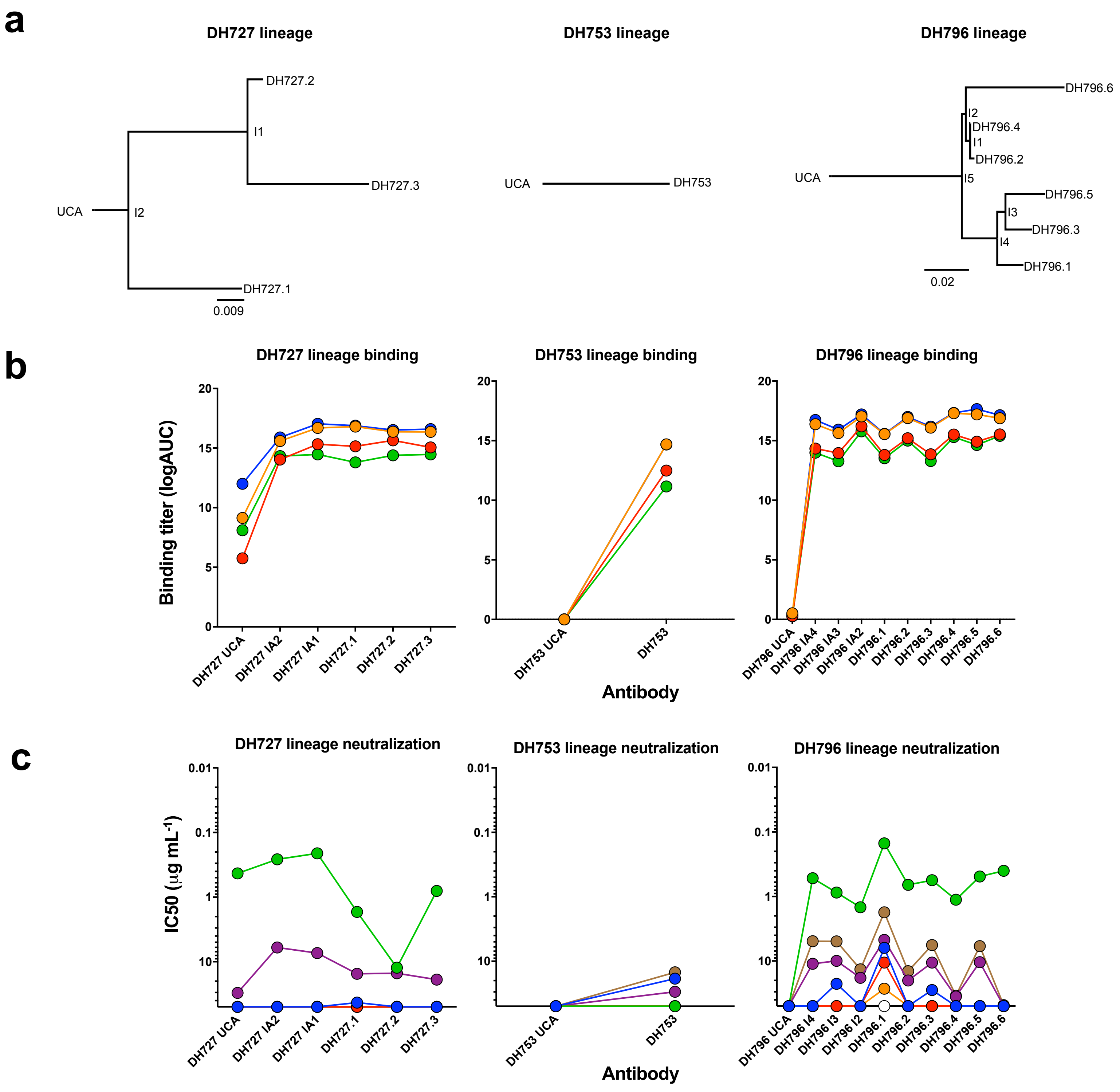

Supplementary Figure 6. Somatic mutation of vaccine-induced antibodies augments Env binding and neutralization.

(a) Maximum-likelihood phylogenetic tree of each antibody lineage was inferred with Cloanalyst. Each tree is rooted with an unmutated common ancestor (UCA).

(b) Env binding titers as log area-under-the-curve (AUC) for each member of the DH727, 753, and 796 antibody lineages. Different color curves show binding titers for different gp120 proteins or V3 region peptides (CH505 TF gp120, blue; CON-S V3, red; ConC V3, green; and A.92RW020 gp120, orange).

(c) Neutralization of HIV-1 infection of TZM-bl cells by the antibodies comprising the three lineages. Neutralization is represented as IC50 for 6 HIV-1 isolates (398-F1_F6_20, blue; CNE8, green; BJ0X002000.03.2, red; CH119.10, orange; 25710-2.43, purple; X1632-S2-B10, brown and the negative control murine leukemia virus SVA (white). Source data are provided as a Source Data file.

Supplementary Table 1. Immunogenetics of clonally related antibodies in the DH727, 753, and 796 antibody lineages.

Antibody[b]	Immunogen	Immunizations[c]	Ig Heavy Chain[a]				Ig Light Chain[a]			
			VH	JH	VH mutation (%, nt)	HCDR3 Length (aa)	VK	JK	VK mutation (%, nt)	LCDR3 Length (aa)
DH727.1	CH505 TF	4X gp120	1-E*01	4-1*01	3.8	10	2-S20*01	3-2*1	2.1	9
DH727.2	CH505 TF	4X gp120	1-E*01	4-1*01	5.2	10	2-S20*01	3-2*1	2.4	9
DH727.3	CH505 TF	6X gp120	1-E*01	4-1*01	8.3	10	2-S20*01	3-2*1	4.3	9
DH753	CON-S	2XDNA/1XrAd5/ 15Xgp140	3-W*02	1-1*01	7.5	13	1-F	1-LC1	4.7	11
DH796.1	VRC-A, B, C	2XDNA/1XrAd5	4-I*01	5-1*01	10	12	2-S20*01	3-2*1	2.1	9
DH796.2	VRC-A, B, C	2XDNA/1XrAd5	4-I*01	5-1*01	8.2	12	2-S20*01	3-2*1	0.4	9
DH796.3	VRC-A, B, C	2XDNA/1XrAd5	4-I*01	5-1*01	10	12	2-S20*01	3-2*1	2.8	9
DH796.4	VRC-A, B, C	2XDNA/1XrAd5	4-I*01	5-1*01	7.9	12	2-S20*01	3-2*1	0.4	9
DH796.5	VRC-A, B, C	2XDNA/1XrAd5	4-I*01	5-1*01	12	12	2-S20*01	3-2*1	2.1	9
DH796.6	VRC-A, B, C	2XDNA/1XrAd5	4-F*01	5-1*01	7.7	12	2-S20*01	3-2*1	2.5	9

a Immunogenetics were inferred with the macaque library in Cloanalyst

b Antibodies were isolated as natural antibody pairs by single B cell PCR.

c DH753 was isolated from a macaque immunized with recombinant adenovirus serotype 5 (rAd5) expressing uncleaved gp140 oligomers and uncleaved gp140 oligomers as recombinant protein. DH796.1 was elicited with DNA immunization encoding gp145 and rAd5 encoding uncleaved gp140.

Supplementary Table 2. Data collection and refinement statistics (molecular replacement)

	DH727.2 + ZAM18 V3 peptide	DH753 + ZAM18 V3 peptide	DH753 + MN V3 peptide
**Data collection**			
Space group	$P12_11$	$P2_12_12_1$	$P12_11$
Cell dimensions			
a, *b*, *c* (Å)	41.51, 95.78, 58.05	45.86, 71.82, 134.75	46.92, 138.32, 71.68
α, β, γ (°)	90, 92.67, 90	90, 90, 90	90, 91.55, 90
Resolution (Å)	50-1.8 (1.83-1.80)	50-2.20 (2.24-2.20)	50-2.70 (2.75-2.70)
R_{sym} or R_{merge}			
$I / \sigma I$	16.7 (3.2)	27.4 (3.1)	17.6 (3.0)
Completeness (%)	99.4 (99.4)	100 (99.9)	99.5 (100)
Redundancy	3.9 (3.6)	7.9 (7.2)	4.0 (4.2)
**Refinement**			
Resolution (Å)	29.0-1.80 (1.85-1.80)	43.4-2.2 (2.26-2.20)	38.77-2.70 (2.77-2.70)
No. reflections	41254 (2726)	23313 (1624)	24977 (1816)
R_{work} / R_{free}	18.46/22.51 (20.74/24.88)	19.19/25.45 (25.19/31.76)	16.99/25.63 (23.65/34.44)
No. atoms			
Protein	3260	3217	6461
Ligand/ion	94	121	231
Water/solvent	269	88	15
B-factors			
Protein	26.15	45.59	70.09
Ligand/ion	38.88	54.05	85.31
Water/solvent	32.20	42.54	83.36
R.m.s. deviations			
Bond lengths (Å)	0.008	0.009	0.009
Bond angles (°)	1.204	1.129	1.245

Each dataset was integrated and scaled from diffraction images collected on one isomorphous crystal.
*Values in parentheses are for highest-resolution shell.

Supplementary Table 3. Rhesus macaque antibody PCR primers.

PCR round	Ig chain	Primer name	Primer sequence
First	Heavy	RHIGVH1+7_EXT1	CACCATGGACTGGACCTGGAGGMTCCTC
First	Heavy	RHIGVH1+7_EXT2	CACCATGGACCTGACCCGGAGGATCCTTTTC
First	Heavy	RHIGVH2_EXT	CACCATGGACACGCTTTGCTCCACRCTC
First	Heavy	RHVH3_EXT1_N	CATGGAGTTGGGGCTGAGCTGGGTYTTCC
First	Heavy	RHVH3_EXT2_N	CATGGAGTTGGGGCTGAGYTGGGTTTTCC
First	Heavy	RHVH3_EXT3_N	CATGGAGTTTGGGCTGAGCTGGR
First	Heavy	RHVH3_EXT4_N	CATGGAGTTTGGGCTGAGCTKGGTTTTYC
First	Heavy	RHVH4_EXT1_N	ACCATGAAGCACCTGTGGTTCTBCCTCCTCC
First	Heavy	RHVH4_EXT2_N	CACCATGAAGCACCTGKGGTTCTTY
First	Heavy	RHIGVH5_EXT	CACCATGGGGTCAACTGCCMTCCTC
First	Heavy	RHIGVH6_EXT	CCATGTCTGTCTCCTTCCTCATCGTCC
First	Heavy	RhIgA_EXTV5	GAAGAAGCCCTGGACCAGGCAGGC
First	Heavy	RhIgG-EXTV5	AAGGTGTGCACGCCGCTGGTCAG
First	Heavy	RhIgM_EXTV5	GTCGGGAAGGAAGTCCTGTGCGAGG
First	Heavy	RhIgD_EXTV5	TCCCCAGGTGCCAGGTGACAGTCAC
First	Heavy	RhIgE_EXTV5	ACGGTCAGCAAGCTGATGGTGGCA
First	Kappa	RHIGVK1_EXT1_N	CACCATGGACATGAGGGYCCC
First	Kappa	RHIGVK1_EXT2_N	CACCATGGACATGAGGGTCCCCAGTC
First	Kappa	RHIGVK1_EXT3_N	CACCATGGACATGAGGGTCCCCGGTTATC
First	Kappa	RHIGVK1_EXT4_N	CACCATGGACATGAGGGTCYCCGGTCAG
First	Kappa	RHIGVK1_EXT5_N	CACCATGGACATGAGGGTCCCCGGTCAGCTYC
First	Kappa	RHIGVK2_EXT1_N	CACCATGAGGCTCCCWGCTCAG
First	Kappa	RHIGVK2_EXT2_N	CACCATGAGGCTCCCTGCTCAGCTYCTGGGGC
First	Kappa	RHIGVK3_EXT1_N	CACCATGGAAGCCCCAGCTCRGCTTCTC
First	Kappa	RHIGVK3_EXT2_N	CACCATGGAAGCCCCAGCACAGCTTCTC
First	Kappa	RHIGVK4_EXT	CACCATGGTGTCACAGACCCAAGWCTTC
First	Kappa	RHIGVK5_EXT	CACCATGGGATCCCAGGTTCACCTCCTCAG
First	Kappa	RHIGVK6_EXT1	CACCATGGTGTCCCCATTGCAACTCCTG
First	Kappa	RHIGVK6_EXT2	CACCATGTTGTCTCCATCACAACTCATTG
First	Kappa	RHIGVK7_EXT	CACCATGGGGTCCTGGGCTCCTTTCCTG
First	Kappa	RhCK_EXTV7	ACCTGATCCTCAGATGGCGGGAAGATG
First	Lambda	RHIGVL1_EXT1	CACCATGGCCTGGTCTCCTCTCSTCCTCAC
First	Lambda	RHIGVL1_EXT2	CACCATGGCCTGGTCTCCTCTCCTTCTC
First	Lambda	RHIGVL2_EXT	CACCATGGCCTGGGCTCTGSTCCTC
First	Lambda	RHIGVL3_EXT1	CACCATGGCCGGGACCCYTCTCCTCCTC
First	Lambda	RHIGVL3_EXT2_N	CACCATGGCCTGGACCCCTGTTCTGCTC

First	Lambda	RHIGVL3_EXT3	CACCATGGCCTGGACCCCTCCCCTRCTC
First	Lambda	RHIGVL4_EXT	CACCATGGCCTGGACCCCACTCCTCCTC
First	Lambda	RHIGVL5_EXT	CACCATGGCCTGGACTCYTCTC
First	Lambda	RHIGVL6_EXT	CACCATGGCCTGGGCTCCACTCCTCCTC
First	Lambda	RHIGVL7_EXT	CACCATGGCCTGGACTCTGCTCCTCCTCC
First	Lambda	RHIGVL8_EXT	CACCATGGCCTGGATGATGCTTCTCCTCG
First	Lambda	RHIGVL11_EXT	CACCATGGCCCTGACTCCTCTCCTCCTC
First	Lambda	RhCL_EXTV7	TGCCATCTGCCTTCCAGGCCACTT
Second	Heavy	RHIGVH1+7_INT1	CCAAGCTGGCTAGCACCATGGACTGGACCTGGAGGMTCCTC
Second	Heavy	RHVH1+7_INT2_N	CCAAGCTGGCTAGCACCATGGACCTSACCCGGAGSATCCTTTTC
Second	Heavy	RHIGVH2_INT	CCAAGCTGGCTAGCACCATGGACACGCTTTGCTCCAC
Second	Heavy	RHVH3_INT1_N	CCAAGCTGGCTAGCACCATGGAGTTGGGGCTGAGYTG
Second	Heavy	RHVH3_INT2_N	CCAAGCTGGCTAGCACCATGGAGTTTGGGCTGAGCTKG
Second	Heavy	RHVH4_INT_N	CCAAGCTGGCTAGCACCATGAAGCACCTGKGGTTC
Second	Heavy	RHIGVH5_INT	CCAAGCTGGCTAGCACCATGGGGTCAACTGCCMTCCTC
Second	Heavy	RHIGVH6_INT	CCAAGCTGGCTAGCACCATGTCTGTCTCCTTCCTCATCGTC
Second	Heavy	RhIgA_ACD_IntV20	CAGGGCCGCTGTGCTCTCGGAGGTGCTCCTGCCCTCGAGGCTCAGCGGGAAGAC
Second	Heavy	RhIgA_BC_IntV20	CAGGGCCGCTGTGCTCTCGGAGGTGCTCCTGCTGCAGAGGYTCAGCGGGAAGAC
Second	Heavy	RhIgG_IntV20	CAGGGCCGCTGTGCTCTCGGAGGTGCTCCTGGAG
Second	Heavy	RhIgM_IntV20	CAGGGCCGCTGTGCTCTCGGAGGTGCTCCTCTCACAGGAGACGAGGGGGAAAAG
Second	Heavy	RhIgD_IntV20	CAGGGCCGCTGTGCTCTCGGAGGTGCTCCTGTTATCCTTTGGGAGTTGGCACGCTG
Second	Heavy	RhIgE_IntV20	CAGGGCCGCTGTGCTCTCGGAGGTGCTCCTGCAGCAGGGGATCAAGGGGAAGAC
Second	Kappa	RHIGVK1_INT1_N	CCAAGCTGGCTAGCACCATGGACATGAGGGYCCC
Second	Kappa	RHIGVK1_INT2_N	CCAAGCTGGCTAGCACCATGGACATGAGGGTCYCCG
Second	Kappa	RHIGVK2_INT1_N	CCAAGCTGGCTAGCACCATGAGGCTCCCWGCTC
Second	Kappa	RHIGVK3_INT1_N	CCAAGCTGGCTAGCACCATGGAAGCCCCAGCWC
Second	Kappa	RHIGVK4_INT	CCAAGCTGGCTAGCACCATGGTGTCACAGACCCAAG
Second	Kappa	RHIGVK5_INT	CCAAGCTGGCTAGCACCATGGGATCCCAGGTTCACCTCC
Second	Kappa	RHIGVK6_INT1	CCAAGCTGGCTAGCACCATGGTGTCCCCATTGCAACTC
Second	Kappa	RHIGVK6_INT2	CCAAGCTGGCTAGCACCATGTTGTCTCCATCACAACTC
Second	Kappa	RHIGVK7_INT	CCAAGCTGGCTAGCACCATGGGGTCCTGGGCTCCTTTCC
Second	Kappa	RhCK_INTv6	TGGCGGGAAGATGAAGACAGATGGTG
Second	Lambda	RHIGVL1_INT	CCAAGCTGGCTAGCACCATGGCCTGGTCTCCTCTC
Second	Lambda	RHIGVL2_INT	CCAAGCTGGCTAGCACCATGGCCTGGGCTCTGSTCC
Second	Lambda	RHIGVL3_INT1_N	CCAAGCTGGCTAGCACCATGGCCGGGACCCYTC

Second	Lambda	RHIGVL3_INT2_N	CCAAGCTGGCTAGCACCATGGCCTGGACCCCTS
Second	Lambda	RHIGVL4_INT_N	CCAAGCTGGCTAGCACCATGGCCTGGACCCCACTCC
Second	Lambda	RHIGVL5_INT	CCAAGCTGGCTAGCACCATGGCCTGGACTCYTCTC
Second	Lambda	RHIGVL6_INT_N	CCAAGCTGGCTAGCACCATGGCCTGGGCTCCACTC
Second	Lambda	RHIGVL7_INT	CCAAGCTGGCTAGCACCATGGCCTGGACTCTGCTCCTC
Second	Lambda	RHIGVL8_INT	CCAAGCTGGCTAGCACCATGGCCTGGATGATGCTTCTC
Second	Lambda	RHIGVL11_INT	CCAAGCTGGCTAGCACCATGGCCCTGACTCCTCTCCTC
Second	Lambda	RhCL_INTv6	GTCACTGATCAGACACACTAGTGTGG

Supplementary references

1. deCamp A, et al. Global panel of HIV-1 Env reference strains for standardized assessments of vaccine-elicited neutralizing antibodies. Journal of virology 88, 2489-2507 (2014).
