# Supplementary material for: Difficult-to-neutralize global HIV-1 isolates are neutralized by antibodies targeting open envelope conformations
Source: Nat Commun. 2019 Jul 1;10:2898. doi: 10.1038/s41467-019-10899-2 (PMC6602974; doi:10.1038/s41467-019-10899-2)
Supplement: Supplementary file 2 — Description of Additional Supplementary Files [file 41467_2019_10899_MOESM2_ESM.docx]

**Description of Supplementary Files**

**File Name:** **Supplementary Data 1.**

**Description:** Vaccine-induced antibody neutralization breadth against 292 HIV-1 pseudoviruses. IC50 and IC80 neutralization titers for vaccine-elicited antibodies DH727.2, DH753, and DH796.1 and HIV-infection-induced antibodies 3074 and 447-52D are shown for each virus. The neutralization titers are color-coded green, light green, yellow, orange, or red in the order of increasing neutralization potency. Clade, recombinant clade, or circulating recombinant form (CRF) is shown for each virus. T/F denotes known transmitted/founder viruses. NA – not applicable as the experiment was not done.

**File Name:** **Supplementary Data 2.**

**Description:** Vaccine-induced antibody neutralization activity across a spectrum of 101 tier 2 HIV-1 pseudoviruses. IC50 neutralization titers for vaccine-elicited antibodies DH727.2, DH753, and DH796.1 are shown for each virus as concentration (µg mL-1). The neutralization titers are color-coded as in Supplementary Data File 1 based on neutralization potency. Viruses are ordered based on sensitivity to 205 individual polyclonal sera from which a geometric mean titer (GMT) was calculated. The GMT is shown as reciprocal plasma dilution that inhibits 50% of virus replication in TZM-bl cells (ID50).
